# Pathophysiological Underpinnings of Extra-Motor Neurodegeneration in Amyotrophic Lateral Sclerosis: New Insights From Biomarker Studies

**DOI:** 10.3389/fneur.2021.750543

**Published:** 2022-01-18

**Authors:** David Reyes-Leiva, Oriol Dols-Icardo, Sonia Sirisi, Elena Cortés-Vicente, Janina Turon-Sans, Noemi de Luna, Rafael Blesa, Olivia Belbin, Victor Montal, Daniel Alcolea, Juan Fortea, Alberto Lleó, Ricard Rojas-García, Ignacio Illán-Gala

**Affiliations:** ^1^Neuromuscular Diseases Unit, Department of Neurology, Hospital de la Santa Creu i Sant Pau, Universitat Autònoma de Barcelona, Barcelona, Spain; ^2^Centro de Investigación Biomédica en Red de Enfermedades Raras, CIBERER, Valencia, Spain; ^3^Sant Pau Memory Unit, Department of Neurology, Biomedical Research Institute Sant Pau, Hospital de la Santa Creu i Sant Pau, Universitat Autònoma de Barcelona, Barcelona, Spain; ^4^Centro de Investigación Biomédica en Red de Enfermedades Neurodegenerativas, CIBERNED, Madrid, Spain

**Keywords:** amyotrophic lateral sclerosis (ALS), neuroimage, cerebrospinal fluid (CSF), biomarker (BM), neuropathology, TDP-43 = TAR DNA-binding protein 43, frontotemporal lobar degeneration, frontotemporal dementia (FTD)

## Abstract

Amyotrophic lateral sclerosis (ALS) and frontotemporal lobar degeneration (FTLD) lie at opposing ends of a clinical, genetic, and neuropathological continuum. In the last decade, it has become clear that cognitive and behavioral changes in patients with ALS are more frequent than previously recognized. Significantly, these non-motor features can impact the diagnosis, prognosis, and management of ALS. Partially overlapping neuropathological staging systems have been proposed to describe the distribution of TAR DNA-binding protein 43 (TDP-43) aggregates outside the corticospinal tract. However, the relationship between TDP-43 inclusions and neurodegeneration is not absolute and other pathophysiological processes, such as neuroinflammation (with a prominent role of microglia), cortical hyperexcitability, and synaptic dysfunction also play a central role in ALS pathophysiology. In the last decade, imaging and biofluid biomarker studies have revealed important insights into the pathophysiological underpinnings of extra-motor neurodegeneration in the ALS-FTLD continuum. In this review, we first summarize the clinical and pathophysiological correlates of extra-motor neurodegeneration in ALS. Next, we discuss the diagnostic and prognostic value of biomarkers in ALS and their potential to characterize extra-motor neurodegeneration. Finally, we debate about how biomarkers could improve the diagnosis and classification of ALS. Emerging imaging biomarkers of extra-motor neurodegeneration that enable the monitoring of disease progression are particularly promising. In addition, a growing arsenal of biofluid biomarkers linked to neurodegeneration and neuroinflammation are improving the diagnostic accuracy and identification of patients with a faster progression rate. The development and validation of biomarkers that detect the pathological aggregates of TDP-43 *in vivo* are notably expected to further elucidate the pathophysiological underpinnings of extra-motor neurodegeneration in ALS. Novel biomarkers tracking the different aspects of ALS pathophysiology are paving the way to precision medicine approaches in the ALS-FTLD continuum. These are essential steps to improve the diagnosis and staging of ALS and the design of clinical trials testing novel disease-modifying treatments.

## Introduction: Using Biomarkers to Reveal the Extra-Motor Underpinnings of Amyotrophic Lateral Sclerosis

Amyotrophic lateral sclerosis (ALS) is a progressive paralytic disorder defined by the neurodegeneration of motor neurons, while frontotemporal dementia (FTD) is the most common presentation of frontotemporal lobar degeneration (FTLD) and is characterized by progressive neurodegeneration of frontotemporal structures (1, 2). The diagnosis of ALS is based on the identification of motor signs and symptoms, while the diagnosis of FTD is based on cognitive, language, and behavioral features (3–5). However, recent evidence indicates that ALS can show varying degrees of cognitive and behavioral changes at diagnosis (6–9). In ALS, the presence of non-motor symptoms has been linked to frontotemporal neurodegeneration (10, 11), and current diagnostic criteria acknowledge the existence of a continuum of cognitive and behavioral changes between ALS without cognitive impairment (ALSno-cbi) and full-blown FTD (12). However, the exact mechanisms driving extra-motor neurodegeneration in ALS remain largely unknown. Filling this gap in our knowledge is essential to improve the diagnosis and management of patients with ALS and may also impact end-of-life legal decisions (13, 14).

From a clinical perspective, ALS and FTLD are the two heterogeneous diseases characterized by multiple clinical presentations and highly variable rates of functional decline (15, 16). The observed phenotypic and prognostic heterogeneity hampers the diagnosis and the prediction of disease progression at the single-subject level and the design of disease-modifying treatments (15). These problems underscore the importance of developing precise, objective, and reproducible biomarkers to improve the diagnosis, prognosis, and disease monitoring (17–19). A biomarker can be defined as objective, quantifiable characteristics of a biological process (either physiological or pathological) that can be objectively measured *in vivo* (20). Biomarkers have proven to be powerful tools to increase diagnostic certainty and predict disease progression in other neurodegenerative diseases (21). Biomarkers also have the potential to reveal the critical aspects of ALS pathophysiology and advance the field toward the implementation of precision medicine approaches (22).

Despite the cumulative evidence suggesting that ALS and FTLD lie on a clinical, pathological, and genetic continuum, the pathophysiological underpinnings of this continuum remain largely unexplored (10). This review aims to (i) summarize the clinical and pathophysiological correlates of extra-motor neurodegeneration in ALS; (ii) review the diagnostic and prognostic value of biomarkers and their potential to characterize extra-motor neurodegeneration in ALS; and (iii) discuss how biomarkers could improve the diagnosis and classification of ALS.

## Extra-Motor Involvement in ALS

### Clinical Evidence for Extra-Motor Involvement in ALS

Although the first observation of the clinical overlap between ALS and FTD dates from the beginning of the twentieth century (1, 7–9, 23–26), the study of this clinical continuum has been boosted in the last decade using the emerging neuropathological, molecular, and genetic evidence connecting the two processes (10, 27–29).

#### Neuropsychological Evidence

The development of specific tools to assess the cognitive status of ALS patients with variable degrees of motor impairment has led to a major breakthrough for the study of the ALS-FTD continuum (26, 30, 31). When studied with specifically designed instruments, such as the Edinburgh ALS cognitive and behavioral screen (ECAS), the ALS cognitive behavioral screen, or the Arrows and Colors Cognitive Test, up to 20% of patients with ALS can also be diagnosed with FTD, and up to 50% of patients with ALS have a cognitive or behavioral impairment (ALScbi) (6, 10, 32–36). The frequency and characteristics of cognitive and behavioral impairment resemble those noted in FTD, and recent neuropathological studies have reported frontotemporal involvement consistent with the diagnosis of FTLD in more than 30% of patients with ALS (37). Among the most common cognitive symptoms developed in patients with ALS-FTD are executive dysfunction, language impairment, and social cognition, all of which are linked to the frontal and temporal lobes (6, 33). Some neuropsychological tools (i.e., *Sydney Language Battery, Test for Reception of Grammar*) have proven to be useful for characterizing the language performance in patients within the ALS-FTD clinical continuum. The presence of language impairment is almost universal in patients meeting the criteria for ALS-FTD. On the other hand, patients with ALS who do not meet the criteria for ALS-ci or ALS-FTD may show only subtle language deficits (38–46). From a behavioral perspective, frontal behaviors like apathy, stereotypic behavior, and disinhibition are the most common behavioral features in patients with ALS (12, 47). However, behavioral changes in patients with ALS include subtle changes in social cognition. These changes can be adequately measured with specific tests such as the *Reading the mind in the eyes test* or *the faux-pas test* (41, 48). On the other hand, up to 15% of patients initially diagnosed with FTD may also develop motor neuron signs during follow-up (10, 49). It should be noted that the frequency of motor neuron signs in patients with FTD is probably underestimated because these signs are not always systematically investigated during follow-up in FTD cohorts.

Neuropsychological testing has proven to be helpful for the clinical characterization of patients with ALS, but neuropsychological evaluations are time-consuming and may not be well-tolerated by some patients. These limitations reinforce the need for the biomarkers that help us to trace the development of extra-motor manifestations (30, 35).

#### Clinical Evidence

As we will discuss in Section 2.3, the neuropathological correlates of cognitive and behavioral changes in ALS are still a matter of debate (6, 12, 33, 47, 49). Several studies have consistently noted that patients with ALS and having a bulbar onset of symptoms have an increased risk of cognitive and behavioral impairment (6, 32, 50). Distal upper-limb weakness, sometimes with no upper motor neuron signs, is also frequently observed in patients meeting the criteria for ALS-FTD (51). Other reports link the development of bulbar symptoms during the disease course with cognitive and behavioral symptoms (52, 53). Interestingly, other studies have also reported a higher presence of abnormal oculomotor findings, extrapyramidal signs, and autonomic dysfunction in patients with ALS and having prominent bulbar symptoms at disease onset (54).

### Genetic Determinants of Extra-Motor Involvement

#### Major Genetic Findings Associated With Extra-Motor Impairment in ALS

Considerable progress has been made in unraveling the genetics of ALS and FTLD, and it is now clear that the genetics of these two neurodegenerative conditions overlap significantly (55). Table 1 summarizes the main characteristics of genes associated with both ALS and FTD etiology. These genes shed some light on several pathophysiological processes, which are relevant for extra-motor involvement in ALS. For instance, the genes involved in the lysosomal function and autophagy process may have a prominent role in both sides of the spectrum and other neurodegenerative diseases (56).

**Table 1:** Genes in close association with both amyotrophic lateral sclerosis (ALS) and frontotemporal dementia (FTD) etiologies.

**Gene**	**Location**	**Pathway**	**MI**
*C9orf72*	9p21.2	Nucleocytoplasmic transport/splicing	AD
*TBK1*	12q14.1	Autophagy/inflammation	AD
*TARDBP*	1p36.2	Nucleocytoplasmic transport/splicing	AD
*SQSTM1*	5q35.3	Autophagy	AD/Risk
*VCP*	9p13.3	Autophagy/mitochondrial function	AD
*FUS*	16p11.2	Nucleocytoplasmic transport/splicing	AD/AR
*UBQLN2*	Xp11	Autophagy/proteasome	XL
*CHCHD10*	22q11.2	Mitochondrial dysfunction/synaptic integrity	AD

*MI, mode of inheritance; AAO, age at onset; DM, disease modifier; AD, autosomal dominant; AR, autosomal recessive; XL, X-linked*.

The *C9orf72* hexanucleotide GGGGCC repeat intronic expansion is the most common genetic cause of both ALS and FTLD. Some noteworthy studies reported that patients with ALS and carrying *C9orf72* have a 4–5-fold higher risk of presenting with cognitive or behavioral changes than non-carriers (57). Physiologic *C9orf72* protein is normally localized in the nuclei, and it regulates membrane trafficking. On the other hand, the hexanucleotide repeat expansion is cytotoxic. While the precise molecular mechanisms underlying cytotoxicity are not fully understood, several mechanisms have been proposed, including a loss of function through haploinsufficiency, RNA toxic gain-of-function through the sequestration and accumulation of toxic dipeptide repeat proteins (DRP) (10, 58–63). As noted in Table 1, other genes causing both ALS and FTLD are related to protein trafficking, cytoskeletal dynamics, protein degradation, and mitophagy (12, 64). Similarly, genes such as *TANK-binding kinase-1* (*TBK1*) (65–69), Sequestosome-1 or p62 (*SQTSM1*) (70), Optineurine protein (*OPTN*), and Valosin-containing protein (*VCP*) that are associated with FTD, inclusion body myositis, motor neuron disease, and Paget's disease encode proteins related to protein degradation (71, 72). In addition, recent studies have suggested that the apolipoprotein E (*APOE*) gene may play an important role in the development of cognitive and behavioral impairment in ALS. The ε4 allele of the *APOE* gene is a major genetic factor in Alzheimer's disease and has also been shown to increase the risk of TAR DNA-binding protein 43 (TDP-43) proteinopathy and hippocampal sclerosis in a large community-based cohort. These results are in line with previous evidence suggesting that the ε4 allele of the *APOE* gene may also increase the risk of FTLD. Interestingly, recent evidence from well-characterized cohorts of ALS showed that the ε2 allele of the *APOE* gene (together with the presence of *C9orf72* repeat expansion) significantly increases the risk of FTD in participants with ALS (73–76). Recent studies on the evaluation of the impact of Hungtingtin (HTT) pathologic expansions in the ALS-FTD spectrum suggest an ethiopathological link (77). Similarly, rare variants in the *GBA* gene encoding glucocerebrosidase (previously described as a potential risk factor of cognitive impairment in Parkinson's disease) are also overrepresented in patients with FTD-ALS and ALScbi compared to patients with ALS and having no cognitive impairment (78).

A recent transcriptome-based study of postmortem frontal cortex tissue from patients with ALS reported a coordinated upregulation of transcripts from a sub-population of microglia (termed disease-associated microglia) and overexpressed neuroinflammatory molecules, such as YKL 40 (also known as CHI3L1) and CHI3L2. Thus, microglial and inflammatory pathways are involved in the pathogenesis of extra-motor impairment in ALS (79).

In summary, genes associated with both ALS and FTD point toward the involvement of pathophysiological processes outside the motor regions that are relevant for extra-motor involvement in patients with ALS.

#### Genetic Findings Associated With Extra-Motor Sparing in ALS

It should be noted that the overlap in genes associated with ALS and FTLD is not complete. For example, superoxide dismutase 1 (*SOD1*), *FUS*, and *TARDBP* variants are most commonly associated with ALS and only rarely cause FTLD. Similarly, *GRN* is linked to the TDP-43 subtype of FTLD but not ALS (80). Interestingly, the reduced expression of GRN has been recently identified as a possible contributing factor in the development of different neurodegenerative diseases including ALS (81). In addition, experiments performed in animal models have identified the reduced expression of GRN as a sufficient factor to induce TDP-43 deposition (82). The gene encoding *SOD1* is rarely related to extra-motor impairment in ALS albeit that some mutations have been recently associated with an early cognitive impairment (83). *SOD1* is essential for antioxidant defense in the cytosol and mitochondria (84). Non native formations of *SOD1* have been detected in small granular *SOD1*-immunoreactive inclusions in the motor neurons of sporadic ALS patients without pathogenic *SOD1* variants and in patients carrying other ALS-associated genes (85, 86). These findings suggest that the misfolding of *SOD1* can be part of a joint downstream event in motor neuron neurodegeneration.

Finally, neuropathological findings in sporadic patients with ALS and having FUS aggregates resemble those of patients with FTLD and having FUS deposition (87). However, mutations in the *FUS* gene are related to ALS and rarely cause FTLD (88). To date, the reasons behind the relative sparing of extra-motor regions in patients with ALS and having *FUS* mutations remain unclear.

Interestingly, some genes that are strongly associated with FTLD have scarcely been studied in the ALS-FTD spectrum, where their role remains unclear. This is the case for *MAPT* mutations, which cause the FTLD tau subtype, and GRN and TREM2 mutations, which have been associated with the TDP-43 subtype and Alzheimer's disease (89–91).

### Neuropathological Underpinnings of Extra-Motor Neurodegeneration

#### Neuropathological Changes in ALS

From a pathological perspective, TDP-43 aggregation is a frequent pathological feature of FTLD and the most typical neuropathological finding in patients with ALS (92–96). The pathological hallmarks of TDP-43 proteinopathies include mislocalization from the nucleus to the cytoplasm, deposition of ubiquitinated and hyperphosphorylated TDP-43 into inclusion bodies, protein truncation leading to the formation of toxic C-terminal TDP-43 fragments, and protein aggregation (97). ALS and FTLD with TDP-43 inclusions can be subdivided into different subtypes based on the anatomical distribution and morphology of abnormal TDP-43 aggregates. *Type A* is observed in patients with *GRN* mutations and is characterized by round “compact” intracytoplasmic TDP-43 inclusions, dystrophic short neurites, and occasional lenticular intranuclear inclusions, mainly in the upper cortical layers. *Type B* is the most frequent subtype in patients with motor neuron diseases and shows many granular TDP-43 inclusions with a few dystrophic neurites in all the cortical layers. *Type C* shows long dystrophic neurites and a few intracytoplasmic inclusions. Finally, *type D* histopathology is associated with a mutation in VCP, which causes familial inclusion body myositis, Paget's disease of bone, FTD with or without motor neuron disease involvement (98–100). Lee et al. reported a new histopathologic subtype for TDP-43 aggregates (*type E*) with specific TDP-43 aggregates, a uniform biochemical profile, and a rapid clinical course (101).

**Figure 1:**
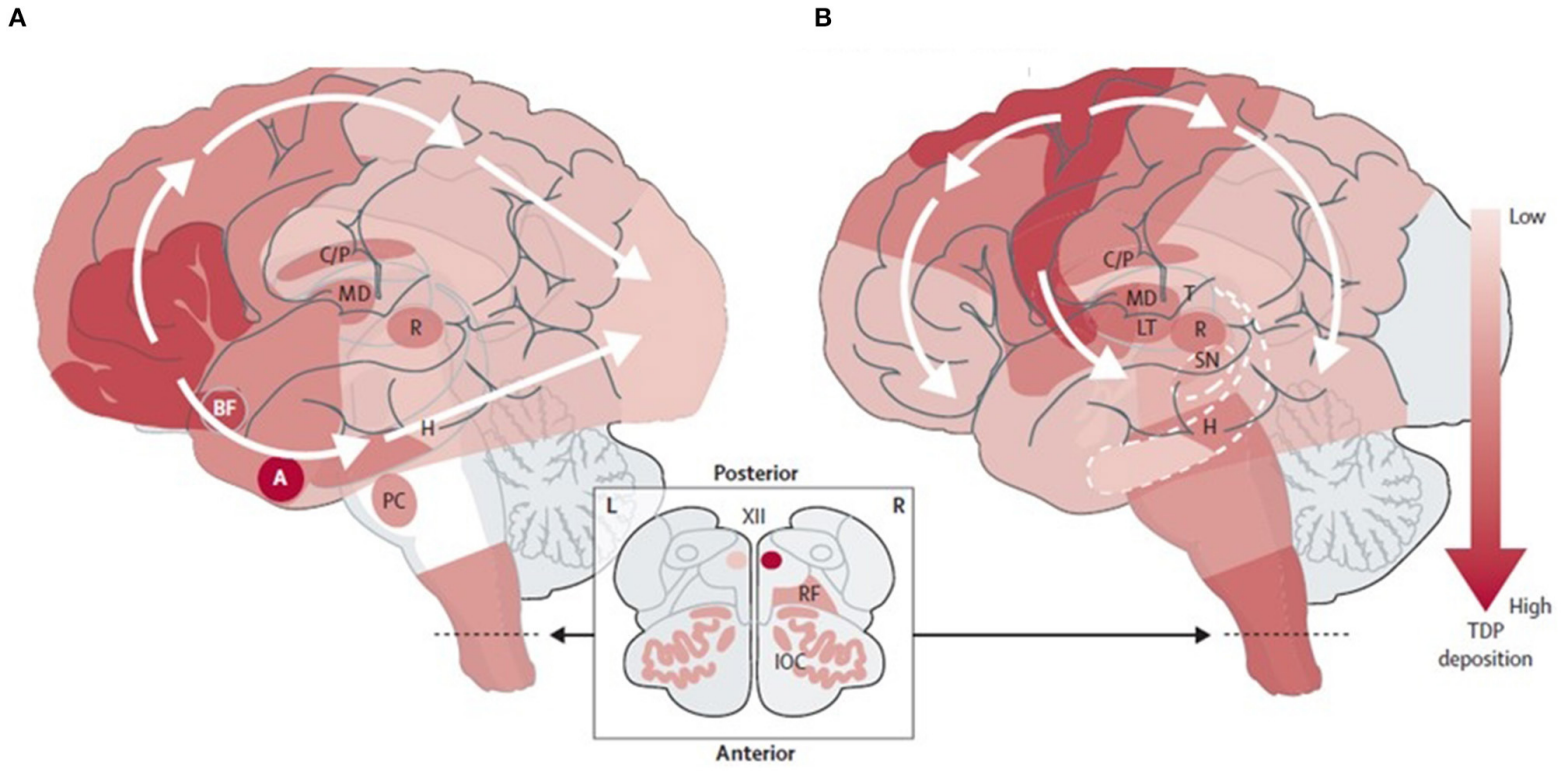
Neuropathological staging schemes in: **(A)** FTLD-TDP (104) and **(B)** ALS-TDP (93). Adapted from Burrell et al. (10). FTLD-TDP, frontotemporal lobar degeneration with TAR DNA-binding protein inclusions; ALS-TDP, amyotrophic lateral sclerosis with TAR DNA-binding protein inclusions. BF, basal forebrain; A, Amygdala; H, Hippocampus; PC, precerebellar nuclei; C/P, Caudate nucleus and putamen; MD, Mediodorsal nucleus of the thalamus; T, Thalamus; LT, Lateral thalamus; R, Red nucleus; SN, Substantia nigra; RF, Reticular formation; IOC, Inferior olivary complex; XII, Hypoglossal nucleus.

Misfolded TDP-43 propagation is believed to follow a “prion-like” mechanism, seeding of the native protein misfolding *in vitro* and synaptic transmission to the next neuron (93, 94, 102–105). This protein deposition is associated with neuronal and synapse loss and affects the different brain regions in ALS and FTD, at least initially. In patients with ALS, the phosphorylated TDP-43 inclusions spread initially from motor neurons located in the motor cortex, spinal cord, and brainstem motor nuclei to other neocortical areas, cerebellum, and striatum (93). In contrast, in FTLD with TDP-43 inclusions, this spread begins in the amygdala and orbitofrontal cortex, spreading to the temporal and frontal non-motor cortex first and motor cortex later, and finally reaching the visual cortex (10, 93–95) (Figure 1). Most studies have shown moderate TDP-43 inclusions before synapse and neuronal loss in animal models (94); however, an intriguing minority of neurons may lack detectable nuclear TDP-43 despite the apparent absence of a cytoplasmic TDP-43 inclusion. These cells show neuronal atrophy comparable to inclusion-bearing neurons, suggesting that the loss of nuclear TDP-43 function promotes neurodegeneration even when TDP-43 aggregation is inconspicuous or absent (106).

#### Neuropathological Correlates of Cognitive and Behavioral Changes in ALS

Pathological aggregates of TDP-43 in extra-motor cortical and subcortical areas are believed to play an essential role in cognitive impairment in ALS. The burden of pathological TDP-43 pathology in extra-motor regions is higher in patients with ALScbi and ALS-FTD than those with ALSno-cbi (39, 107, 108). Also, synapse loss has been identified using high-resolution imaging of postmortem brain tissue samples in extra-motor cortical areas, such as the prefrontal cortex of sporadic patients with ALS (109). Notably, the degree of synapse loss correlated with the severity of the patients' cognitive impairment independent of cortical atrophy, supporting the idea that synapse loss precedes neuronal loss (109, 110). Finally, Tau pathology and hippocampal sclerosis may also play a secondary role in the presence of cognitive impairment in patients with ALS (37, 111, 112).

#### Neuroinflammation

Neuroinflammation in ALS and FTD is primarily characterized by the activation of innate immune sensing pathways in microglia and astrocytes resident to the central nervous system but also with the involvement of peripheral-derived inflammatory cells, such as T-lymphocytes, mast cells, monocyte-derived macrophages, and dendritic cells (113–116). As part of the innate immune system, some components of the innate complement system have been identified in postmortem studies and their eventual role in disease progression (117, 118). Also, microglial density and microglial activation measured using immunostaining for glial fibrillary acidic protein (GFAP) and CD6, show an increase in patients with ALS and FTD and correlate well with synaptic, axonal, and neuronal loss and faster motor and extra-motor disease progression (119–121). There is also a higher presence of reactive astrocytes, which are believed to have neurotoxic properties and have been described involving the gray matter of motor and non-motor cortex in patients with ALS and also in affected cortical areas in FTD (122–124). In addition, oligodendrocytes pathology mediated by phosphorylated TDP-43 deposition in postmortem tissue is a common finding in multiple cortical and subcortical areas of patients with ALS, but its significance remains uncertain (125, 126). Taken together, these findings highlight the importance of concurring neuroinflammatory processes in the pathophysiology of both ALS and FTD.

#### Other Neuropathological Hallmarks of ALS: C9orf72, FUS-FET

As aforementioned (*refer to Genetic determinants of extra-motor features*), patients carrying C9orf72 mutation have been associated with more cognitive impairment and more extensive TDP-43 deposition following predominantly a Type B or a Type A + B pattern (93, 127). A p62 and ubiquitin negative intracytoplasmic and intranuclear inclusions corresponding to dipeptide repeat are found in extra-motor areas, such as the frontal cortex, hippocampus, and cerebellum (12, 62, 110, 128). Interestingly, some reports have also identified *C9orf72* patients with ALS and FTD presenting with cognitive impairment but with minimally TDP-43 inclusions underscoring the influence of other physiopathological mechanisms such as dipeptide repeat accumulation and RNA foci in the appearance of cognitive impairment (63, 108).

Conversely, in the case of FUS protein deposition neuropathology, it is believed that the pathological processes underlying the aggregate formation and cell death between FTD and ALS differ. While ALS with *FUS* mutations seems to be more restricted to the dysfunction of FUS protein, a more complex dysregulation that includes the deposition of all FET proteins (TAF15 and EWS) and Transportin-1 (Trn1) is thought to be involved in the subtypes of FTLD with FUS pathology (88).

## Biomarkers

Biomarkers can be used for four primary purposes: (i) to guide clinical diagnosis by identifying the key pathophysiological changes of a particular disease (so-called diagnostic markers), (ii) to estimate the risk or speed of progression of a particular disease (prognostic markers), (iii) to monitor the progression or response to therapy (theragnostic markers) (129), and (iv) to characterize the relevant aspects of disease pathophysiology (i.e., inflammation). Section Fluid Biomarkers will describe the currently available biomarkers for the study of extra-motor neurodegeneration in the ALS-FTLD continuum and their diagnostic and prognostic value.

### Fluid Biomarkers

The cerebrospinal fluid (CSF) represents an invaluable sample for studying neurodegenerative diseases due to its close relationship with brain parenchyma (130). However, the study of CSF biomarkers may be limited because of their invasive nature. Recently, blood biomarkers have shown promise as minimally invasive clinical tools in neurodegenerative diseases (129, 131). Compared to CSF, the accessibility and cost-effectiveness of blood samples make them more suitable for first-line clinical use and facilitate clinical trial recruitment and monitoring.

#### Neurodegeneration Biomarkers

Neurofilaments are the principal constituents of the neuro-axonal cytoskeleton and play an essential part in axonal transport and the synapse (132). Neurofilament light chain (NfL) is the most abundant and soluble neurofilament subunit, and increased levels in CSF and blood reflect axonal damage in the CNS. The CSF and plasma levels of NfL are highly correlated and have been found to increase in a wide variety of diseases (133, 134). Both CSF and plasma levels of NfL are increased in patients with ALS and have proven helpful to differentiate between patients with ALS and other ALS mimics (135–137). Importantly, multimodal biomarker studies have shown that the CSF and plasma levels of NfL correlate well with frontotemporal cortical thickness and white matter microstructure in patients within the ALS-FTD continuum (138–140). The CSF and blood levels of NfL also correlate with measures of disease severity, disease progression rate, and the prediction of longitudinal clinical deterioration and survival (21, 137, 138, 141–148). The CSF NfL levels seem to increase over time but further longitudinal data are needed (149, 150). Moreover, the combination of plasma NfL with other measurements of neuroinflammation in plasma may improve disease progression at the single-subject level (151–153). Because biofluid levels of NfL are strongly correlated with age, recent multicenter studies have determined highly specific age-adjusted cut-offs (154). Overall, both CSF and plasma levels of NfL may be helpful to support the diagnosis in clinically relevant scenarios, disease staging, and the prediction of disease progression. In addition to NfL, neurofilament heavy chain (NfH) has also been studied in patients with ALS and can discriminate between ALS, ALS mimics, and healthy controls with similar accuracy to NfL (155). Of note, a recent study showed that the difference in the abundance of NfH between ALS and FTD was markedly stronger for NfH than that of NfL (155). On the other hand, studies have reported a better correlation between the serum and CSF for NfL compared to NfH, albeit that NfL has been more widely investigated (156). Overall, CSF NfL and NfH as well as serum NfL are equally suited for the differential diagnosis of ALS, whereas serum NfH performs less well (155). Other serum biomarkers, such as peptides linked with eating behavior and metabolism, for instance, leptin and neuropeptide-Y, have been recently studied in the ALS-FTD continuum and show a promise as neurodegeneration biomarkers, but further investigations are needed to clarify their role in the pathophysiology of ALS and FTD (157).

Patients within the ALS-FTD clinical continuum also show increased levels of total tau (t-tau) in CSF but the diagnostic and prognostic value of this biomarker is lower than NfL (145, 158). Some studies have reported that low levels of both phosphorylated tau at threonine 181 (p-tau) and the p-tau:t-tau ratio in CSF may differentiate TDP-43 and tau subtypes of FTLD (159–162), but these findings seem to be driven by the higher levels of t-tau in patients with ALS (162–164) and not by lower levels of p-tau in FTLD-TDP (165).

#### Synapse Degeneration Markers

The amyloid precursor protein (APP) is a type I single-pass transmembrane protein with a large extracellular domain and a short cytoplasmic tail that undergoes very complex proteolytic processing, yielding biologically active fragments (166). These include the full-length Aβ1–42 and other N-terminal and C-terminal truncated forms of Aβ, sAPPα, sAPPβ, etc., that can be detected in human CSF (167–169). Numerous studies suggest that reduced neuronal-synaptic activity may also lead to less Aβ production (170, 171) and that Aβ may also have an important role in synaptic function (172). Recent studies on autopsy-confirmed cases have reported reduced CSF concentrations of sAPPβ in FTLD in the absence of comorbid Alzheimer's pathology (173). Interestingly, CSF concentrations of sAPPβ are decreased in ALS and FTD compared to healthy controls and correlate with frontotemporal cortical thickness and cognitive impairment suggesting that sAPPβ levels may reflect the neuronal loss in frontotemporal areas (140, 174). Unfortunately, plasma concentrations of sAPPβ poorly correlate with CSF concentrations (175), potentially due to a confounding effect of abundant non-CNS-derived sAPPβ in the blood. On the other hand, CSF concentrations of neurogranin, the most studied synaptic marker to date, were not elevated in patients with ALS (176). In another study, label-free mass spectrometry-based proteomic analyses of CSF from patients with ALS revealed a concomitant decrease in proteins from biological pathways related to synapse organization and inflammation-related proteins (177). Recently, other synaptic markers have been identified by unbiased proteomics-based approaches. Still, more studies are needed to clarify the role of these new biomarkers and their potential use for the diagnosis, staging, and prognosis of patients with suspected ALS (178, 179).

#### Biomarkers Linked to Neuroinflammation and Glial Activation

A variety of systemic inflammatory responses (pro- and anti-inflammatory cytokine profiles, altered immune cell populations) have been consistently reported in patients with ALS and FTD (113, 180). These responses do not seem to be a mere passive phenomenon and may contribute to the neurodegeneration/neuroprotection balance (181, 182). For instance, increased CSF and serum levels of some monocyte-derived cytokines linked with the inflammatory response such as TNF-α, IL-1β, IL-17, and TGF-β reveal a microglial and white blood cell participation in the ALS-FTD pathologic continuum (113, 182, 183). In addition, increased CSF levels compared to controls of Matrix Metalloproteinase-10 and decreased CSF levels of some chemokines and growth factors (such as IL-8, IL-12B, TGF-α among others) have been recently reported in a multicentric study (184). Other surrogate markers of acute-phase systemic inflammation have been reported in ALS, including lipopolysaccharide-binding protein, C reactive protein, and soluble CD14 (181, 185).

In addition to a systemic inflammatory response, several biomarkers in the blood and CSF have been found to be consistently altered in the ALS-FTD continuum (186). Some of these biomarkers may be useful to predict disease progression and survival (140, 187). Increased CSF levels of YKL-40 (also known as Chitinase like-3 protein 1) in ALS were first observed in a small study (188), a finding that has been later replicated in larger studies involving participants along the ALS-FTD continuum (140). Recent proteomic studies confirmed that three macrophage-derived chitinases (YKL-40, chitotriosidase, and chitinase like-3 protein 2) were increased in ALS and predicted a faster disease course but only YKL-40 levels increased over time in those with low initial levels (187). These results support a crucial role for microglial activity in ALS (152), offering novel target engagement and pharmacodynamic biomarkers for neuroinflammation-focused ALS therapy. However, YKL-40 is not specific to the central nervous system, and their levels in the CSF and blood are poorly correlated, thus limiting its use as a non-invasive biomarker. Conversely, GFAP is a brain-specific protein and an established marker of astrogliosis. This protein can also be accurately measured in the blood with new ultrasensitive technology (189). Interestingly, different neuroinflammatory profiles between ALS and FTD have been recently reported, with increased CSF levels of the chitinase chitotriosidase 1 in ALS and high levels of GFAP in FTD (190).

The microglial transmembrane receptor (TREM2) represents another important molecule linked to microglial activity. One study reported that the *TREM2 p.R47H* genetic variant may represent a risk factor for sporadic ALS (191), but this finding has not been replicated in subsequent studies (192, 193). In addition, the soluble part of the microglial transmembrane receptor (sTREM2) is measurable in the blood and CSF, but the information regarding its role within the ALS-FTD continuum is scarce (194). Finally, immune innate complement factors involved in the inflammatory process have been studied in the CSF of patients with ALS finding an upregulation of C3 in CSF and increased levels of C3 cleavage products in the serum of patients with ALS (195, 196).

#### Biomarkers Linked to Abnormal TDP-43 Aggregation

Cytoplasmic aggregation of ubiquitinated, phosphorylated, and truncated TDP-43 is a unifying pathologic observation across the neuropathological continuum of ALS and FTLD. However, standard immunoassays of TDP-43 have not proven to be useful to identify patients with pathological TDP-43 aggregation, and it is not yet clear whether TDP-43 would be expected to increase in the CSF due to the degeneration of nerve cells and the release of intracellular proteins or whether it would be expected to be low in the CSF due to aggregate formation (197–199). This is because most currently available techniques for quantifying TDP-43 use commercially available antibodies, which only detect the full-length form of TDP-43 rather than the disease-specific forms of TDP-43 (200, 201). However, several recent advances hold promise to the detection of abnormal TDP-43 aggregation *in vivo*. A recent study used immuno-IR sensors to study the secondary structure of all TDP-43 isoforms in the CSF (202). In addition, a recent study showed that real-time quaking-induced conversion reaction (RT-QuIC) antemortem prion detection was a robust technique for prion amplification of TDP-43 (203).

Some promising biomarkers like truncated Stathmin-2 (STMN2) are linked to TDP-43 function and deposition. The expression of STMN2 is increased in the frontal cortex of patients with the TDP subtype of FTLD, which makes it a good candidate biomarker related to TDP pathology (204). Finally, in C9orf72 carriers, high CSF levels of DRPs can be detected even in pre-symptomatic stages, suggesting a role of DRPs early in ALS-FTD pathogenesis in early stages (145, 205). Although not currently relevant as clinical fluid biomarkers, DRPs may become more widely used in detecting the *C9orf72* expansion prior to genetic screening or in prodromal stages, which would make a valuable addition to the biomarker arsenal in the future clinical trials.

### Image-Based Biomarkers

#### Macrostructural and Microstructural Changes in MRI

**Figure 2:**
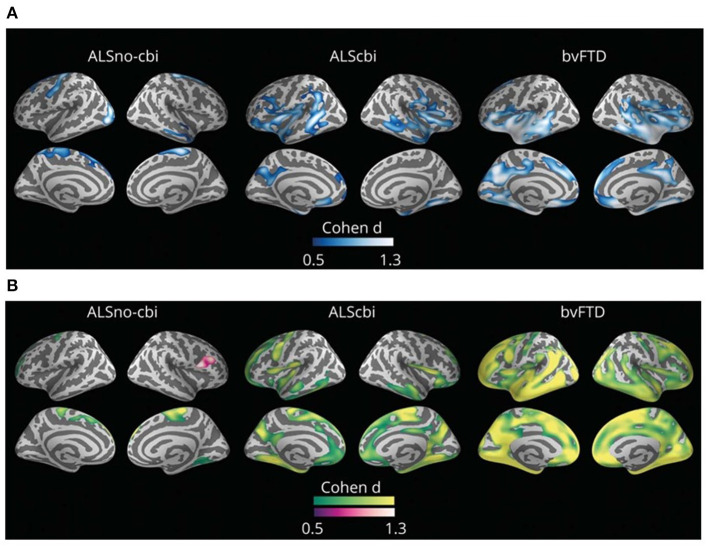
Group comparisons between those with amyotrophic lateral sclerosis (ALS) without cognitive or behavioral impairment (ALSno-cbi; left column), those with ALS with cognitive or behavioral impairment (ALScbi; middle column), and those with the behavioral variant of frontotemporal dementia (bvFTD; right column) compared to controls for **(A)** cortical thickness and **(B)** cortical mean diffusivity. Regions in blue represent thinner cortex; regions in green represent higher cortical mean diffusivity; and regions in purple represent lower cortical mean diffusivity. All analyses were adjusted for age, sex, education, and MRI equipment. Only clusters that survived family-wise error correction *p* < 0.05 are shown. ALSno-cbi, Amyotrophic Lateral Sclerosis without cognitive and behavioral impairment; ALScbi, Amyotrophic Lateral Sclerosis with cognitive and behavioral impairment; bvFTD, Behavioral variant of frontotemporal dementia.

Routine structural MRI has a limited utility in ALS as signal intensity, and gross volume changes in T1- and T2-weighted images are not typically observed in most ALS cases. On the contrary, quantitative analysis of MRI can improve the detection of neurodegeneration-related abnormalities by measuring small changes in cerebral structure. The *in vivo* quantification of motor and extra-motor cortical cerebral changes with MRI analyses is of great value in monitoring disease progression in the ALS-FTD continuum (Figure 2) (206–208). In sporadic ALS cases, recent meta-analyses of neuroimaging studies report consistent gray matter loss in the precentral gyrus, inferior frontal gyrus, cingulate/paracingulate gyrus, and rolandic operculum (209, 210). This cortical thinning in extra-motor cortical areas correlates with cognitive and behavioral impairment in the neuropsychological test (211, 212). On the other hand, *C9orf72* cases and a subgroup of sporadic cases without the *C9orf72 expansion* are characterized by widespread cortical changes characterized by cortical thinning involving bilateral pars opercularis, fusiform, lingual and parietal cortex, and also smaller volumes in the right hippocampus and bilateral thalamus (213, 214).

Interestingly, Dadar et al. were able to replicate the neuropathological stages of TDP-43 *in vivo* with a novel neuroimaging technique (215). However, some neuroimaging methods, which quantify brain atrophy (i.e., gray matter loss or cortical thickness), show limited sensitivity to the earliest cortical changes related to cortical dysfunction (158, 216). Diffusion tensor imaging (DTI) is a diffusion weighted imaging sequence that measures the diffusion of water molecules in the different directions of space (217). The main advantage of DTI is that it is able to detect subtle changes at the microstructural level reflecting the breakdown of biological barriers and neuroinflammation (218, 219). Thus, DTI studies allow the study of microstructural changes of the cerebral cortex (i.e., cortical mean diffusivity) and the white matter (i.e., microstructural changes in different white matter tracts). Several studies on neurodegenerative diseases, including FTD, have shown that DTI can reveal pathology that is not detected with cortical thickness measurements (216, 219–223). A recent cross-sectional study showed that ALScbi displayed cortical mean diffusivity changes in extra-motor regions in the absence of cortical thinning and showed a better correlation with cognition than with cortical thickness. These results suggest that cortical mean diffusivity may be more sensitive than cortical thickness to detect extra-motor cortical changes in ALS, but more studies are needed to characterize the longitudinal changes of cortical microstructure in ALS (158). On the other hand, longitudinal studies using DTI to study the white matter microstructure in ALS have shown the usefulness of this approach to detect extra-motor changes and monitor disease progression (222, 224–226).

#### Functional Magnetic Resonance

fMRI based on the measurements of the fluctuation in blood flow and blood oxygen level as a consequence of neuronal activity helps us to trace the underlying networks between the areas that coactivate simultaneously. Assessing the resting-state brain connectivity in patients with ALS has revealed either decreased or increased connectivity in premotor, sensorimotor, and basal ganglia (227, 228). Advanced network-based neuroimaging techniques based on connectomics and a whole-brain connectivity analysis have shown different patterns of motor and extra-motor involvement between different MND phenotypes (229).

#### Magnetic Resonance Spectroscopy

MRS provides a means of measuring cerebral metabolites relevant to neurodegeneration *in vivo* [for a review, Kalra et al. (230), Ernst et al. (231)]. In ALS, neurochemical changes reflecting neuronal loss or dysfunction (i.e., decreased N-acetylaspartate) are mostly significant in the motor cortex and corticospinal tracts. Other neurochemical changes observed include increased myo-inositol, a putative marker of gliosis. Some studies also reported cerebral metabolite changes in extra-motor regions. Previous studies performed in asymptomatic genetic carriers of mutations associated with FTLD using MRS-derived markers exhibit an encouraging discriminatory ability to identify patients from healthy controls; however, more data are needed to determine their ability to assist with the diagnosis in early stages and in distinguishing from disease mimics (232–239).

#### PET: 18F-Fluorodeoxyglucose Radiotracer

PET represents a powerful imaging biomarker for the study of neurodegenerative dementias (240, 241). An increasing number of 18F-labeled tracers are not available for use at the clinical site without the requirement of an on-site cyclotron, thus turning brain PET scans into a widely applicable tool. Brain hypometabolism can be detected with PET and the 18F-fluorodeoxyglucose radiotracer (18F-FDG-PET). Of note, 18F-FDG-PET imaging has some advantages over MRI as it can be performed in patients needing mechanical ventilation. Cerebral metabolism reflects the local intensity of brain glutamatergic synaptic and astrocyte activity, and cerebral hypometabolism is considered an indirect marker of neurodegeneration (240, 242). In ALS, previous studies investigating 18F-FDG-PET revealed a marked frontal and prefrontal relative hypometabolism in ALScbi when compared to patients with ALSno-cbi (243, 244). Unexpectedly, previous studies also reported hypermetabolism in the midbrain, temporal poles, and hippocampus (245). It has been hypothesized that hypermetabolism in patients with ALS may be related to microglial activity in these areas but more multimodal biomarker studies are needed to understand the pathophysiological underpinnings of cortical and subcortical metabolism in ALS (206, 245, 246). Overall, previous studies support the view that 18F-FDG-PET could be a useful tool to assess the spread of brain pathology *in vivo*.

#### PET to Detect Neuroinflammation

Multiple imaging studies have reported *in vivo* microglial activation in motor and extra-motor regions in both ALS and FTD using different radiotracers with affinity to the translocator protein or TSPO (which is expressed in the mitochondria of activated microglia) (247–250). Nevertheless, some caveats of those radiotracers might influence their usefulness; the most used radiotracer 11C-PK11195, which labels peripheral benzodiazepine binding sites, has a low signal-to-background ratio and binding of the subsequent generation of radiotracers (11C-PBR28, 18F-DPA714, and 18F-FEPPA) is influenced by the TSPO polymorphism (247, 250–252). Thus, more work is needed for the validation of radiotracers that can detect and monitor microglial activation *in vivo*.

#### PET to Detect TDP-43 Pathology

The development of a radiotracer able to bind phosphorylated TDP-43 and allow the *in vivo* visualization of pathological TDP inclusions would be a major breakthrough in the field. Unfortunately, such a biomarker is not available at this moment (251).

## Other Biomarkers

### Neurophysiological Biomarkers

Neurophysiological studies have shown that hyperexcitability of the frontotemporal cortex is an early feature of ALS that may precede motor neuron degeneration and can be assessed using transcranial magnetic stimulation with single or multiple pulses (253). Transcranial magnetic stimulation with multiple pulses evaluating the short-interval intracortical inhibition is one of the most robust tests developed for assessing cortical hyperexcitability and has shown good sensitivity and specificity for ALS diagnosis and a good correlation with other biomarkers of peripheral neurodegeneration (254, 255). Recent studies have demonstrated a more prominent cortical hyperexcitability in patients with ALS presenting with cognitive symptoms than in cognitively unimpaired patients with ALS (256). Hyperexcitability in the sensory cortex may induce increased amplitudes that can be detected early in ALS and correlate with a shorter disease survival (257). Some groups have tried to assess cortical connectivity and the interaction between the motor and non-motor network using other techniques, such as electroencephalography and fMRI, but the clinical utility of these techniques is still limited (258, 259).

### Genetic Biomarkers

MicroRNAs are short (about 22 nucleotides in length) non-coding RNA molecules that play a relevant role as endogenous regulators of gene expression (260). Emerging studies have shown that circulating microRNAs can serve as potential biomarkers in ALS. However, there are still many problems to be solved before microRNAs could be used in clinical practice [for a detailed review please refer to Wang and Zhang (261)].

## Discussion: Advancing ALS Classification With Biomarkers

Phenotypic classification of ALS relies on clinical observations such as site and region of onset and predominance of upper or lower motor neuron signs, while the diagnosis of FTD is based on clinical criteria (2, 24). Although ALS and FTD share partially overlapping patterns of neurodegeneration, the generation of clinically defined subtypes is not straightforward due to the observed clinical and prognostic heterogeneity. The definition of precise and reproducible classification systems for both ALS and FTD is urgently needed to guide present and future treatments, provide an indication of prognosis and enable the analysis in clinical trials of homogenous groups for a more personalized approach to therapy (24, 262). These refined classification systems would ideally consider the etiology (i.e., proteinopathy responsible for the patient's symptoms), other clinical, or biological features for robust disease staging at the single-subject level (i.e., scales measuring a motor or cognitive function, or frontotemporal atrophy burden on neuroimage) and finally the quantification of other pathophysiological processes that are relevant for prognosis (i.e., microglial activation at diagnosis). In the following section, we will discuss how biomarkers (alone or in combination with multimodal biomarkers studies) could be integrated into multidimensional classification systems to further improve the diagnosis and prognosis of these complex neurodegenerative diseases.

### Etiology: Early Identification of TDP-43 Proteinopathies

There is no definitive diagnostic test for ALS or due to abnormal TDP-43 aggregation (1). Because clinical-pathological correlations are imperfect, biomarkers, which can detect abnormal TDP-43 aggregates *in vivo*, are needed to provide an early etiological diagnosis (i.e., ALS caused due to abnormal TDP-43 vs. FUS or tau aggregates in FTD). Recently developed RT-QuIC assays for TDP-43 are promising (203). Concomitant TDP-43 pathology can also be seen in a significant number of patients with Alzheimer's disease neuropathological changes, as well as in a smaller proportion of other neurodegenerative diseases (263–268). Thus, an understanding of how abnormal TDP-43 aggregates impact brain function may also be valuable to understanding its role in other neurodegenerative dementias. Of note, pathological TDP-43 aggregates are a key neuropathological finding in the recently described neuropathological entity named “Limbic predominant Age-Related TDP-43 pathology encephalopathy” or simply “LATE” (269). This new neuropathological construct may account for up to 22% of the attributable risk of developing dementia of the Alzheimer's type and has shown to impact the clinical presentation and prognosis in patients with Alzheimer's disease neuropathological changes. However, it is unclear whether “LATE” is the underlying pathology in cases with the FTLD TDP-43 subtype (263, 270).

A recent study on neuropathologically confirmed FTLD suggests the existence of disparate patterns of gray and white matter involvement in FTLD subtypes, with more prominent involvement of white matter in tauopathies than in TDP-43 proteinopathies (271). We envision that the combination of cortical mean diffusivity with additional measures of subcortical white matter microstructure and the use of fluid-based biomarkers of neurodegeneration and synaptic loss (i.e., NfL, sAPPb) could reveal important insights into the etiology of neurodegeneration by identifying specific neurodegenerative signatures *in vivo*. This approach may also improve clinical-pathological correlations in some challenging clinical scenarios. For example, it may help identify patients presenting with a non-fluent agrammatic variant of primary progressive aphasia with underlying TDP-43 pathology and possibly benefit from future protein-specific treatments (272).

### Disease Staging: Looking Beyond Corticospinal Tract Neurodegeneration

Imaging studies have consistently reported that extra-motor neurodegeneration in ALS is related to cognitive impairment and that the frequency of cognitive and behavioral impairment seems to increase with disease progression (33). Prefrontal changes detected with MRI *in vivo* predict the progression rate, and recent studies suggest that the topography of extra-motor atrophy in patients with ALS parallels the neuropathological stages of TDP-43 (215). However, neuroimaging studies investigating the structural correlates of cognitive and behavioral impairment have failed to define a consistent topography of extra-motor involvement (210, 212, 273). One limitation of previous studies is the application of neuroimaging techniques that may not capture the earliest cortical changes driving cognitive and behavioral impairment in ALS such as gray matter density or cortical thickness (274). On the contrary, other DTI-based MRI biomarkers have been shown to be sensitive to the earliest neurodegeneration-related changes and may be of great value to monitor disease progression (158, 207). More studies are needed to determine the potential utility of DTI-based imaging techniques for quantifying disease burden within first and second motor neurons and extra-motor neurodegeneration. This information could allow the refinement of current classification systems and advance the field toward a multidimensional approach for the classification of neurodegenerative diseases (275).

### The Relationship Between Astroglial Activity and Disease Progression in the ALS-FTD Continuum

As discussed previously, several biofluid biomarkers have proven to be useful to predict disease progression in patients with ALS. Due to the role of microglial reactivity in the neurodegenerative process, YKL-40 might be useful for the prognostic stratification in the ALS-FTD spectrum (187). The combination of multiple biomarkers, such as CSF cytokines and innate complement system fragments in a single study may provide important clues to understand the role of neuroinflammation in ALS and FTD pathophysiology. However, one important limitation of biofluid biomarkers is that they do not provide information regarding the topography of neurodegeneration (190). A recent study combined structural MRI with the use of a PET ligand to measure microglial activation and showed that the areas of ligand binding were correlated with motor signs, cortical thinning, reduced fractional anisotropy and increased diffusivity (248). Interestingly, this study did not identify a change in ligand binding in subjects undergoing repeat imaging over 6 months despite clinical progression, suggesting that glial activation is present early and does not change at least in terms of TSPO imaging. This study illustrated the potential of multimodal biomarker studies to understand the role of neuroinflammation in ALS. More studies and better tools are needed to clearly define how neuronal-glial signaling occurs at the different stages of the disease, and where potential therapeutic interventions can be made to modulate inflammation to slow disease progression (113).

### Beyond TDP-43: Identifying Fast and Slow Progressors

Although TDP-43 is the major neuropathological hallmark of the disease in most ALS cases, other pathophysiological processes play an important role. Clinical-pathological studies investigating the relationship between pathological aggregates of TDP-43 and cognitive impairment have revealed conflicting results (33, 108). However, a recent study reported that pre-mortem cognitive performance was better correlated with postmortem synaptic density than with postmortem cortical thickness or neuronal loss (109). In addition, cognitive and behavioral changes were better correlated with cortical microstructure than with cortical thickness in a recent neuroimaging study involving patients from the ALS-FTD clinical continuum (158).

Of note, early neurodegenerative changes and synapse loss can be captured with novel radiotracers capturing synaptic density but to date, these novel imaging methods have not been assessed in ALS (276, 277). Taken together, these findings suggest that synaptic loss and other neurodegeneration-related changes may antedate cortical atrophy and pathological aggregates of TDP-43 but additional studies are needed to clarify the relationship between non-motor symptoms, synaptic density, cortical microstructure, and cerebral metabolism. In addition, the exact significance of cortical mean diffusivity changes in ALS is unknown. Future multimodal biomarker studies combining 18F-FDG-PET, cortical mean diffusivity, and measures of microglial activity and neuroinflammation will be helpful to understand the pathophysiology of the earliest microstructural changes in ALS.

**Figure 3:**
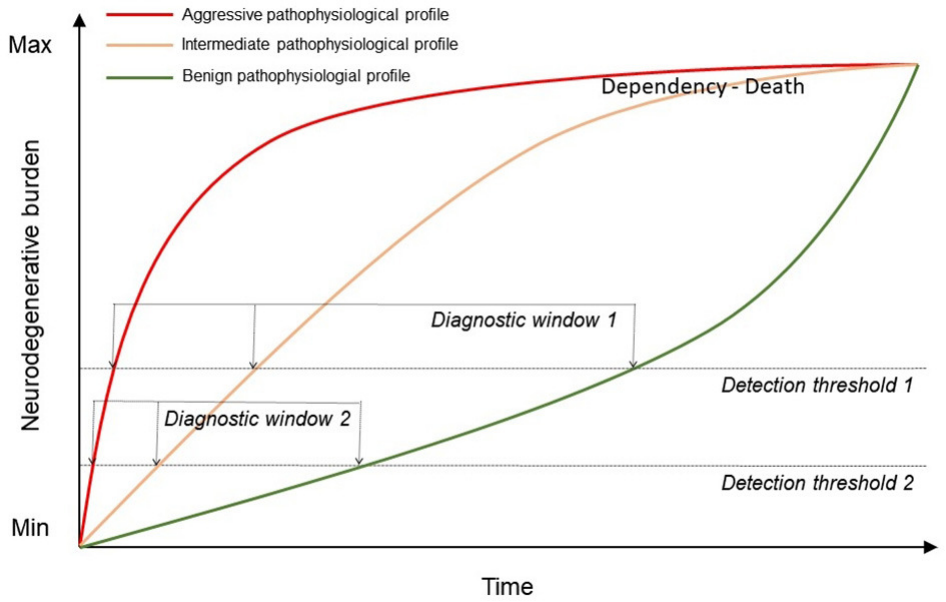
Representation of the increasing neurodegenerative burden in patients with ALS over time depending on their pathophysiological profile. The use of biomarkers may help to improve the current detection threshold (detection threshold and diagnostic window 1) from 1 to earlier clinical stages and differentiating between profiles (detection threshold and diagnostic window 2).

Figure 3 illustrates the potential use of biomarkers to advance the diagnosis of ALS in earlier clinical stages when future targeted therapies may be more effective. The information provided by biomarkers at diagnosis could also be considered for the prediction of disease progression and the selection of candidates for clinical trials. This information may be crucial for the candidate selection for future trials targeting a specific aspect of ALS pathophysiology (i.e., anti-inflammatory drugs). On the other hand, the development of novel imaging biomarkers more sensitive than conventional imaging biomarkers may increase our sensitivity for the detection of FTLD-related neurodegenerative changes by lowering the detection threshold. This, in turn, may allow at diagnosis the earliest clinical stages. Finally, pathophysiological biomarkers measuring the key aspects of the neurodegenerative process (i.e., neuroinflammation or FTLD subtype) could play an important role in future multimodal classification schemes, similar to the recently proposed biomarker-based classification systems for other neurodegenerative diseases (278).

## Conclusions

The fact that some patients diagnosed with ALS develop cognitive and behavioral changes while others remain cognitively intact is intriguing. This observation may provide important clues to the specific vulnerability of the frontotemporal cortex in neurodegenerative dementias. As we have shown in this review, biomarkers have the potential to improve our understanding of the pathophysiological underpinnings of extra-motor neurodegeneration in ALS. Emerging imaging biomarkers have shown a significant potential to improve the diagnosis and staging of patients within the ALS-FTD clinical continuum. In addition, several biofluid biomarkers have the potential to increase the diagnostic certainty of underlying ALS or FTLD, predict disease progression at the single-subject level, and characterize the pathophysiological underpinnings of extra-motor neurodegeneration in ALS. Future studies for determining the benefit of current and future biomarkers to advance ALS classification and monitoring are the essential steps for improving the design of clinical trials testing novel disease-modifying treatments.

## Author Contributions

II-G and DR-L designed and drafted the manuscript. OD-I, EC-V, JT-S, NL, RB, OB, VM, DA, JF, AL, and RR-G provided critical feedback and helped review and edit the manuscript. All authors contributed to the article and approved the submitted version.

## Funding

This study was supported by the Fondo de Investigaciones Sanitario (FIS), Instituto de Salud Carlos III (PI21/00791 to II-G, PI13/01532 and PI16/01825 to RB, PI14/1561 and PI17/01896 to AL, and PI15/01618 and PI19/01543 to RR-G), the CIBERNED program (Program 1, Alzheimer's Disease to AL and SIGNAL study, www.signalstudy.es), and partly jointly funded by Fondo Europeo de Desarrollo Regional, Unión Europea, Una manera de hacer Europa. This work was supported by Departament de Salut de la Generalitat de Catalunya, Pla Estratègic de Recerca i Innovació en Salut (SLT002/16/00408 to AL), Fundació La Marató de TV3 (044412 to RB and 201437.10 to RR-G). II-G was supported by the Juan Rodes grant (JR20/0018) and the Pilot Awards for Global Brain Health Leaders (GBHI ALZ UK-21-720973). This work was also supported by an Alzheimer's Association Grant (AACSF-21-850193).

## Conflict of Interest

The authors declare that the research was conducted in the absence of any commercial or financial relationships that could be construed as a potential conflict of interest.

## Publisher's Note

All claims expressed in this article are solely those of the authors and do not necessarily represent those of their affiliated organizations, or those of the publisher, the editors and the reviewers. Any product that may be evaluated in this article, or claim that may be made by its manufacturer, is not guaranteed or endorsed by the publisher.

## References

[1] Brown RH Al-Chalabi A. Amyotrophic lateral sclerosis. N Engl J Med. (2017) 377:162–72. 10.1056/NEJMra1603471 28700839

[2] Elahi FM Miller BL. A clinicopathological approach to the diagnosis of dementia. Nat Rev Neurol. (2017) 13:457–76. 10.1038/nrneurol.2017.96 28708131 PMC5771416

[3] Brooks BR Miller RG Swash M Munsat TL. El escorial revisited: revised criteria for the diagnosis of amyotrophic lateral sclerosis. Amyotroph Lateral Scler Other Motor Neuron Disord. (2000) 1:293–9. 10.1080/146608200300079536 11464847

[4] Rascovsky K Hodges JR Knopman D Mendez MF Kramer JH Neuhaus J. Sensitivity of revised diagnostic criteria for the behavioural variant of frontotemporal dementia. Brain. (2011) 134:2456–77. 10.1093/brain/awr179 21810890 PMC3170532

[5] Gorno-Tempini ML Hillis AE Weintraub S Kertesz A Mendez M Cappa SF. Classification of primary progressive aphasia and its variants. Neurology. (2011) 76:1006–14. 10.1212/WNL.0b013e31821103e6 21325651 PMC3059138

[6] Goldstein LH Abrahams S. Changes in cognition and behaviour in amyotrophic lateral sclerosis: nature of impairment and implications for assessment. Lancet Neurol. (2013) 12:368–80. 10.1016/S1474-4422(13)70026-7 23518330

[7] Kew JJM Goldstein LH Leigh PN Abrahams S Cosgrave N Passingham RE. The relationship between abnormalities of cognitive function and cerebral activation in amyotrophic lateral sclerosis: a neuropsychological and positron emission tomography study. Brain. (1993) 116:1399–423. 10.1093/brain/116.6.1399 8293278

[8] Abrahams S Goldstein LH Al-Chalabi A Pickering A Morris RG Passingham RE. Relation between cognitive dysfunction and pseudobulbar palsy in amyotrophic lateral sclerosis. J Neurol Neurosurg Psychiatry. (1997) 62:464–72. 10.1136/jnnp.62.5.464 9153602 PMC486852

[9] Lomen-Hoerth C Murphy J Langmore S Kramer JH Olney RK Miller B. Are amyotrophic lateral sclerosis patients cognitively normal? Neurology. (2003) 60:1094–7. 10.1212/01.WNL.0000055861.95202.8D 12682312

[10] Burrell JR Halliday GM Kril JJ Ittner LM Götz J Kiernan MC. The frontotemporal dementia-motor neuron disease continuum. Lancet. (2016) 388:919–31. 10.1016/S0140-6736(16)00737-6 26987909

[11] Neary D Snowden JS Mann DM Northen B Goulding PJ Macdermott N. Frontal lobe dementia and motor neuron disease. J Neurol Neurosurg Psychiatry. (1990) 53:23–32. 10.1136/jnnp.53.1.23 2303828 PMC1014093

[12] Strong MJ Abrahams S Goldstein LH Woolley S Mclaughlin P Snowden J. Amyotrophic lateral sclerosis - frontotemporal spectrum disorder (ALS-FTSD): Revised diagnostic criteria. Amyotrophic Lateral Scler Frontotemporal Degener. (2017) 18:153–74. 10.1080/21678421.2016.1267768 28054827 PMC7409990

[13] Elamin M Bede P Byrne S Jordan N Gallagher L Wynne B. Cognitive changes predict functional decline in ALS: a population-based longitudinal study. Neurology. (2013) 80:1590–7. 10.1212/WNL.0b013e31828f18ac 23553481

[14] Merrilees J Klapper J Murphy J Lomen-Hoerth C Miller BL. Cognitive and behavioral challenges in caring for patients with frontotemporal dementia and amyotrophic lateral sclerosis. Amyotrophic Later Scler. (2010) 11:298–302. 10.3109/17482961003605788 20222805 PMC2908374

[15] Westeneng H-J Debray TPA Visser AE van Eijk RPA Rooney JPK Calvo A. Prognosis for patients with amyotrophic lateral sclerosis: development and validation of a personalised prediction model. Lancet Neurol. (2018) 17:423–33. 10.1016/S1474-4422(18)30089-9 29598923

[16] Illán-Gala I Falgàs N Friedberg A Castro-Suárez S Keret O Rogers N. Diagnostic utility of measuring cerebral atrophy in the behavioral variant of frontotemporal dementia and association with clinical deterioration. JAMA Netw Open. (2021) 4:e211290. 10.1001/jamanetworkopen.2021.1290 33704477 PMC7953307

[17] Woolley JD Khan BK Murthy NK Miller BL Rankin KP. The diagnostic challenge of psychiatric symptoms in neurodegenerative disease: rates of and risk factors for prior psychiatric diagnosis in patients with early neurodegenerative disease. J Clin Psychiatry. (2011) 72:126–33. 10.4088/JCP.10m06382oli 21382304 PMC3076589

[18] Martínez-Molina M Argente-Escrig H Polo MF Hervás D Frasquet M Cortés V. Early referral to an ALS center reduces several months the diagnostic delay: a multicenter-based study. Front Neurol. (2020) 11:604922. 10.3389/fneur.2020.604922 33391167 PMC7775542

[19] Paganoni S Macklin EA Lee A Murphy A Chang J Zipf A. Diagnostic timelines and delays in diagnosing amyotrophic lateral sclerosis (ALS). Amyotroph Lateral Scler Frontotemporal Degener. (2014) 15:453–6. 10.3109/21678421.2014.903974 24981792 PMC4433003

[20] Biomarkers Definitions Working Group. Biomarkers and surrogate endpoints: Preferred definitions and conceptual framework. Clin Pharmacol Ther. (2001) 69:89–95. 10.1067/mcp.2001.113989 11240971

[21] Meeter LH Kaat LD Rohrer JD van Swieten JC. Imaging and fluid biomarkers in frontotemporal dementia. Nat Rev Neurol. (2017) 13:406–19. 10.1038/nrneurol.2017.75 28621768

[22] Jameson JL Longo DL. Precision medicine — personalized, problematic, and promising. N Engl J Med. (2015) 372:2229–34. 10.1056/NEJMsb1503104 26014593

[23] Braumühl AV. Pick's disease and amyotrophic lateral sclerosis. Allgem Zeitschrift Fur Psychiatr Psychol Med. (1932) 96:364–6.

[24] Al-Chalabi A Hardiman O Kiernan MC Chiò A Rix-Brooks B van den Berg LH. Amyotrophic lateral sclerosis: moving towards a new classification system. Lancet Neurol. (2016) 15:1182–94. 10.1016/S1474-4422(16)30199-5 27647646

[25] Bak TH. Learning from history-FTD, ALS and behaviour. Rinsho Shinkeigaku. (2010) 50:1013. 10.5692/clinicalneurol.50.1013 21921548

[26] Woolley SC Strong MJ. Frontotemporal dysfunction and dementia in amyotrophic lateral sclerosis. Neurol Clin. (2015) 33:787–805. 10.1016/j.ncl.2015.07.011 26515622

[27] Al-Chalabi A Jones A Troakes C King A Al-Sarraj S van den Berg LH. The genetics and neuropathology of amyotrophic lateral sclerosis. Acta Neuropathol. (2012) 124:339–52. 10.1007/s00401-012-1022-4 22903397

[28] Ng ASL. Genetics of frontotemporal dementia in Asia: advancing knowledge through collaboration. Neurology. (2015) 85:2060–2. 10.1212/WNL.0000000000002045 26432849

[29] Swinnen B Robberecht W. The phenotypic variability of amyotrophic lateral sclerosis. Nat Rev Neurol. (2014) 10:661–70. 10.1038/nrneurol.2014.184 25311585

[30] Abrahams S Newton J Niven E Foley J Bak TH. Screening for cognition and behaviour changes in ALS. Amyotrophic Later Scler Frontotemporal Degener. (2014) 15:9–14. 10.3109/21678421.2013.805784 23781974

[31] Lillo P Savage S Mioshi E Kiernan MC Hodges JR. Amyotrophic lateral sclerosis and frontotemporal dementia: a behavioural and cognitive continuum. Amyotrophic Later Scler. (2012) 13:102–9. 10.3109/17482968.2011.639376 22214356

[32] Montuschi A Iazzolino B Calvo A Moglia C Lopiano L Restagno G. Cognitive correlates in amyotrophic lateral sclerosis: a population-based study in Italy. J Neurol Neurosurg Psychiatry. (2015) 86:168–73. 10.1136/jnnp-2013-307223 24769471

[33] Crockford C Newton J Lonergan K Chiwera T Booth T Chandran S. ALS-specific cognitive and behavior changes associated with advancing disease stage in ALS. Neurology. (2018) 91:e1370–80. 10.1212/WNL.0000000000006317 30209236 PMC6177274

[34] Niven E Newton J Foley J Colville S Swingler R Chandran S. Validation of the edinburgh cognitive and behavioural amyotrophic lateral sclerosis screen (ECAS): a cognitive tool for motor disorders. Amyotrophic Later Scler Frontotemporal Degener. (2015) 16:172–9. 10.3109/21678421.2015.1030430 25967542

[35] Woolley SC York MK Moore DH Strutt AM Murphy J Schulz PE. Detecting frontotemporal dysfunction in ALS: utility of the ALS cognitive behavioral screen (ALS-CBS^TM^). Amyotrophic Later Scler. (2010) 11:303–11. 10.3109/17482961003727954 20433413

[36] Raaphorst J Beeldman E Schmand B Berkhout J Linssen WHJP van den Berg LH. The ALS-FTD-Q: a new screening tool for behavioral disturbances in ALS. Neurology. (2012) 79:1377–83. 10.1212/WNL.0b013e31826c1aa1 22972650

[37] Borrego-Écija S Turon-Sans J Ximelis T Aldecoa I Molina-Porcel L Povedano M. Cognitive decline in amyotrophic lateral sclerosis: neuropathological substrate and genetic determinants. Brain Pathol. (2021) 31:e12942. 10.1111/bpa.12942 33576076 PMC8412113

[38] Pinto-Grau M Hardiman O Pender N. The study of language in the amyotrophic lateral sclerosis - frontotemporal spectrum disorder: a systematic review of findings and new perspectives. Neuropsychol Rev. (2018) 28:251–68. 10.1007/s11065-018-9375-7 29705950

[39] Gregory JM McDade K Bak TH Pal S Chandran S Smith C. Executive, language and fluency dysfunction are markers of localised TDP-43 cerebral pathology in non-demented ALS. J Neurol Neurosurg Psychiatry. (2020) 91:149–57. 10.1136/jnnp-2019-320807 31515300 PMC6996101

[40] Poletti B Carelli L Faini A Solca F Meriggi P Lafronza A. the arrows and colors cognitive test (ACCT): a new verbal-motor free cognitive measure for executive functions in ALS. PLoS ONE. (2018) 13:e0200953. 10.1371/journal.pone.0200953 30091987 PMC6084851

[41] Baron-Cohen S Wheelwright S Hill J Raste Y Plumb I. The “Reading the mind in the eyes” test revised version: a study with normal adults, and adults with asperger syndrome or high-functioning autism. J Child Psychol Psychiatry. (2001) 42:241–51. 10.1111/1469-7610.00715 11280420

[42] Savage S Hsieh S Leslie F Foxe D Piguet O Hodges JR. Distinguishing subtypes in primary progressive aphasia: application of the Sydney language battery. Dement Geriatr Cogn Disord. (2013) 35:208–18. 10.1159/000346389 23467307

[43] Bishop DV. Comprehension of spoken, written and signed sentences in childhood language disorders. J Child Psychol Psychiatry. (1982) 23:1–20. 10.1111/j.1469-7610.1982.tb00045.x 6174536

[44] Meier SL Charleston AJ Tippett LJ. Cognitive and behavioural deficits associated with the orbitomedial prefrontal cortex in amyotrophic lateral sclerosis. Brain. (2010) 133:3444–57. 10.1093/brain/awq254 20889583

[45] Abrahams S Leigh PN Harvey A Vythelingum GN Grisé D Goldstein LH. Verbal fluency and executive dysfunction in amyotrophic lateral sclerosis (ALS). Neuropsychologia. (2000) 38:734–47. 10.1016/S0028-3932(99)00146-3 10689049

[46] Girardi A MacPherson SE Abrahams S. Deficits in emotional and social cognition in amyotrophic lateral sclerosis. Neuropsychology. (2011) 25:53–65. 10.1037/a0020357 20919762

[47] Strong MJ Yang W. The frontotemporal syndromes of ALS. Clinicopathological correlates. J Mol Neurosci. (2011) 45:648–55. 10.1007/s12031-011-9609-0 21809041

[48] De Marchi F Carrarini C De Martino A Diamanti L Fasano A Lupica A. Cognitive dysfunction in amyotrophic lateral sclerosis: can we predict it? Neurol Sci. (2021) 42:2211–22. 10.1007/s10072-021-05188-0 33772353 PMC8159827

[49] Burrell JR Kiernan MC Vucic S Hodges JR. Motor neuron dysfunction in frontotemporal dementia. Brain. (2011) 134:2582–94. 10.1093/brain/awr195 21840887

[50] Gordon PH Delgadillo D Piquard A Bruneteau G Pradat P-F Salachas F. The range and clinical impact of cognitive impairment in French patients with ALS: a cross-sectional study of neuropsychological test performance. Amyotroph Lateral Scler. (2011) 12:372–8. 10.3109/17482968.2011.580847 21585273

[51] Cortés-Vicente E Turon-Sans J Gelpi E Clarimón J Borrego-Écija S Dols-Icardo O. Distinct clinical features and outcomes in motor neuron disease associated with behavioural variant frontotemporal dementia. Dement Geriatr Cogn Disord. (2018) 45:220–31. 10.1159/000488528 29886477

[52] Gromicho M Kuzma-Kozakiewicz M Szacka K Nieporecki K Andersen PM Grosskreutz J. Motor neuron disease beginning with frontotemporal dementia: clinical features and progression. Amyotrophic Later Scler Frontotemporal Degener. (2021) 1–9. 10.1080/21678421.2021.1910309 34229542

[53] Chiò A Moglia C Canosa A Manera U Vasta R Brunetti M. Cognitive impairment across ALS clinical stages in a population-based cohort. Neurology. (2019) 93:e984–94. 10.1212/WNL.0000000000008063 31409738 PMC6745732

[54] McCluskey L Vandriel S Elman L Van Deerlin VM Powers J Boller A. ALS-Plus syndrome: non-pyramidal features in a large ALS cohort. J Neurol Sci. (2014) 345:118–24. 10.1016/j.jns.2014.07.022 25086858 PMC4177937

[55] Abramzon YA Fratta P Traynor BJ Chia R. The overlapping genetics of amyotrophic lateral sclerosis and frontotemporal dementia. Front Neurosci. (2020) 14:42. 10.3389/fnins.2020.00042 32116499 PMC7012787

[56] Root J Merino P Nuckols A Johnson M Kukar T. Lysosome dysfunction as a cause of neurodegenerative diseases: lessons from frontotemporal dementia and amyotrophic lateral sclerosis. Neurobiol Dis. (2021) 154:105360. 10.1016/j.nbd.2021.105360 33812000 PMC8113138

[57] Sabatelli M Marangi G Conte A Tasca G Zollino M Lattante S. New ALS-related genes expand the *spectrum paradigm* of amyotrophic lateral sclerosis: new ALS-related genes expand the *spectrum paradigm*. Brain Pathol. (2016) 26:266–75. 10.1111/bpa.12354 26780671 PMC8029226

[58] Belzil VV Bauer PO Prudencio M Gendron TF Stetler CT Yan IK. Reduced C9orf72 gene expression in c9FTD/ALS is caused by histone trimethylation, an epigenetic event detectable in blood. Acta Neuropathol. (2013) 126:895–905. 10.1007/s00401-013-1199-1 24166615 PMC3830740

[59] Gendron TF Bieniek KF Zhang Y-J Jansen-West K Ash PEA Caulfield T. Antisense transcripts of the expanded C9ORF72 hexanucleotide repeat form nuclear RNA foci and undergo repeat-associated non-ATG translation in c9FTD/ALS. Acta Neuropathol. (2013) 126:829–44. 10.1007/s00401-013-1192-8 24129584 PMC3830741

[60] Mori K Weng S-M Arzberger T May S Rentzsch K Kremmer E. The C9orf72 GGGGCC repeat is translated into aggregating dipeptide-repeat proteins in FTLD/ALS. Science. (2013) 339:1335–8. 10.1126/science.1232927 23393093

[61] Zu T Liu Y Banez-Coronel M Reid T Pletnikova O Lewis J. RAN proteins and RNA foci from antisense transcripts in C9ORF72 ALS and frontotemporal dementia. Proc Nat Acad Sci USA. (2013) 110:E4968–77. 10.1073/pnas.1315438110 24248382 PMC3870665

[62] Balendra R Isaacs AM. C9orf72-mediated ALS and FTD: multiple pathways to disease. Nat Rev Neurol. (2018) 14:544–58. 10.1038/s41582-018-0047-2 30120348 PMC6417666

[63] Vatsavayai SC Yoon SJ Gardner RC Gendron TF Vargas JNS Trujillo A. Timing and significance of pathological features in *C9orf72* expansion-associated frontotemporal dementia. Brain. (2016) 139:3202–16. 10.1093/brain/aww250 27797809 PMC5790143

[64] Mejzini R Flynn LL Pitout IL Fletcher S Wilton SD Akkari PA. ALS genetics, mechanisms, and therapeutics: where are we now? Front Neurosci. (2019) 13:1310. 10.3389/fnins.2019.01310 31866818 PMC6909825

[65] Gijselinck I Van Mossevelde S van der Zee J Sieben A Philtjens S Heeman B. Loss of TBK1 is a frequent cause of frontotemporal dementia in a Belgian cohort. Neurology. (2015) 85:2116–25. 10.1212/WNL.0000000000002220 26581300 PMC4691687

[66] van der Zee J Gijselinck I Dillen L Van Langenhove T Theuns J Engelborghs S. A Pan- e uropean study of the *C9orf72* repeat associated with FTLD : Geographic prevalence, genomic instability, and intermediate repeats. Hum Mutat. (2013) 34:363–73. 10.1002/humu.22244 23111906 PMC3638346

[67] Dols-Icardo O García-Redondo A Rojas-García R Borrego-Hernández D Illán-Gala I Muñoz-Blanco JL. Analysis of known amyotrophic lateral sclerosis and frontotemporal dementia genes reveals a substantial genetic burden in patients manifesting both diseases not carrying the *C9orf72* expansion mutation. J Neurol Neurosurg Psychiatry. (2018) 89:162–8. 10.1136/jnnp-2017-316820 28889094

[68] Pilli M Arko-Mensah J Ponpuak M Roberts E Master S Mandell MA. TBK-1 promotes autophagy-mediated antimicrobial defense by controlling autophagosome maturation. Immunity. (2012) 37:223–34. 10.1016/j.immuni.2012.04.015 22921120 PMC3428731

[69] Heo J-M Ordureau A Paulo JA Rinehart J Harper JW. The PINK1-PARKIN mitochondrial ubiquitylation pathway drives a program of OPTN/NDP52 recruitment and TBK1 activation to promote mitophagy. Mol Cell. (2015) 60:7–20. 10.1016/j.molcel.2015.08.016 26365381 PMC4592482

[70] Hirano M Nakamura Y Saigoh K Sakamoto H Ueno S Isono C. Mutations in the gene encoding p62 in Japanese patients with amyotrophic lateral sclerosis. Neurology. (2013) 80:458–63. 10.1212/WNL.0b013e31827f0fe5 23303844

[71] Pottier C Rampersaud E Baker M Wu G Wuu J McCauley JL. Identification of compound heterozygous variants in *OPTN* in an ALS-FTD patient from the CReATe consortium: a case report. Amyotrophic Later Scler Frontotemporal Degener. (2018) 19:469–71. 10.1080/21678421.2018.1452947 29558868 PMC6116528

[72] Johnson JO Mandrioli J Benatar M Abramzon Y Van Deerlin VM Trojanowski JQ. Exome sequencing reveals VCP mutations as a cause of familial ALS. Neuron. (2010) 68:857–64. 10.1016/j.neuron.2010.11.036 21145000 PMC3032425

[73] Chiò A Brunetti M Barberis M Iazzolino B Montuschi A Ilardi A. The role of APOE in the occurrence of frontotemporal dementia in amyotrophic lateral sclerosis. JAMA Neurol. (2016) 73:425–30. 10.1001/jamaneurol.2015.4773 26903389

[74] Yang H-S Yu L White CC Chibnik LB Chhatwal JP Sperling RA. Evaluation of TDP-43 proteinopathy and hippocampal sclerosis in relation to APOE ε4 haplotype status: a community-based cohort study. Lancet Neurol. (2018) 17:773–81. 10.1016/S1474-4422(18)30251-5 30093249 PMC6154505

[75] Wennberg AM Tosakulwong N Lesnick TG Murray ME Whitwell JL Liesinger AM. Association of apolipoprotein E ε4 with transactive response DNA-binding protein 43. JAMA Neurol. (2018) 75:1347. 10.1001/jamaneurol.2018.3139 30422173 PMC6248121

[76] Rubino E Vacca A Govone F De Martino P Pinessi L Rainero I. Apolipoprotein E polymorphisms in frontotemporal lobar degeneration: a meta-analysis. Alzheimers Dement. (2013) 9:706–13. 10.1016/j.jalz.2012.10.013 23688578

[77] Dewan R Chia R Ding J Hickman RA Stein TD Abramzon Y. Pathogenic huntingtin repeat expansions in patients with frontotemporal dementia and amyotrophic lateral sclerosis. Neuron. (2021) 109:448–60.e4. 10.1016/j.neuron.2021.04.020 33242422 PMC7864894

[78] Canosa A Grassano M Moglia C Iazzolino B Peotta L Gallone S. GBA variants influence cognitive status in amyotrophic lateral sclerosis. J Neurol Neurosurg Psychiatry. (2021). 10.1136/jnnp-2021-327426. [Epub ahead of print]. 34583942 PMC8921570

[79] Dols-Icardo O Montal V Sirisi S López-Pernas G Cervera-Carles L Querol-Vilaseca M. Motor cortex transcriptome reveals microglial key events in amyotrophic lateral sclerosis. Neurol Neuroimmunol Neuroinflamm. (2020) 7:e829. 10.1212/NXI.0000000000000829 32669313 PMC7371375

[80] Gass J Cannon A Mackenzie IR Boeve B Baker M Adamson J. Mutations in progranulin are a major cause of ubiquitin-positive frontotemporal lobar degeneration. Hum Mol Genet. (2006) 15:2988–3001. 10.1093/hmg/ddl241 16950801

[81] Nalls MA Blauwendraat C Sargent L Vitale D Leonard H Iwaki H. Evidence for *GRN* connecting multiple neurodegenerative diseases. Brain Commun. (2021) 3:fcab095. 10.1093/braincomms/fcab095 34693284 PMC8134835

[82] Zhang J Velmeshev D Hashimoto K Huang Y-H Hofmann JW Shi X. Neurotoxic microglia promote TDP-43 proteinopathy in progranulin deficiency. Nature. (2020) 588:459–65. 10.1038/s41586-020-2709-7 32866962 PMC7746606

[83] Martinelli I Zucchi E Gessani A Fini N Chiò A Pecoraro V. A novel p.N66T mutation in exon 3 of the SOD1 gene: report of two families of ALS patients with early cognitive impairment. Amyotrophic Later Scler Frontotemporal Degener. (2020) 21:296–300. 10.1080/21678421.2020.1746344 32248719

[84] Juneja T Pericak-Vance MA Laing NG Dave S Siddique T. Prognosis in familial amyotrophic lateral sclerosis: progression and survival in patients with glu100gly and ala4val mutations in Cu, Zn superoxide dismutase. Neurology. (1997) 48:55–7. 10.1212/WNL.48.1.55 9008494

[85] Forsberg K Graffmo K Pakkenberg B Weber M Nielsen M Marklund S. Misfolded SOD1 inclusions in patients with mutations in *C9orf72* and other ALS/FTD-associated genes. J Neurol Neurosurg Psychiatry. (2019) 90:861–9. 10.1136/jnnp-2018-319386 30992335 PMC6691870

[86] Forsberg K Jonsson PA Andersen PM Bergemalm D Graffmo KS Hultdin M. Novel antibodies reveal inclusions containing non-native SOD1 in sporadic ALS patients. PLoS ONE. (2010) 5:e11552. 10.1371/journal.pone.0011552 20644736 PMC2904380

[87] Borrego-Écija S Cortés-Vicente E Cervera-Carles L Clarimón J Gámez J Batlle J. Does ALS-FUS without *FUS* mutation represent ALS-FET? Report of three cases. Neuropathol Appl Neurobiol. (2019) 45:421–6. 10.1111/nan.12527 30375034 PMC7380051

[88] Neumann M Bentmann E Dormann D Jawaid A DeJesus-Hernandez M Ansorge O. FET proteins TAF15 and EWS are selective markers that distinguish FTLD with FUS pathology from amyotrophic lateral sclerosis with FUS mutations. Brain. (2011) 134(Pt. 9):2595–609. 10.1093/brain/awr201 21856723 PMC3170539

[89] Zhang C-C Zhu J-X Wan Y Tan L Wang H-F Yu J-T. Meta-analysis of the association between variants in MAPT and neurodegenerative diseases. Oncotarget. (2017) 8:44994–5007. 10.18632/oncotarget.16690 28402959 PMC5546535

[90] Sellami L Rucheton B Ben Younes I Camuzat A Saracino D Rinaldi D. Plasma progranulin levels for frontotemporal dementia in clinical practice: a 10-year French experience. Neurobiol Aging. (2020) 91:167.e1–9. 10.1016/j.neurobiolaging.2020.02.014 32171590

[91] Zhang K Lu Y Chen J Li J Yadav KK Yin J. NEK1 and GRN mutations coexist in a sporadic Chinese Hui descent ALS patient. Amyotroph Lateral Scler Frontotemporal Degener. (2020) 21:624–6. 10.1080/21678421.2020.1779301 32772750

[92] Neumann M Sampathu DM Kwong LK Truax AC Micsenyi MC Chou TT. Ubiquitinated TDP-43 in frontotemporal lobar degeneration and amyotrophic lateral sclerosis. Science. (2006) 314:130–3. 10.1126/science.1134108 17023659

[93] Brettschneider J Del Tredici K Toledo JB Robinson JL Irwin DJ Grossman M. Stages of pTDP-43 pathology in amyotrophic lateral sclerosis: ALS stages. Ann Neurol. (2013) 74:20–38. 10.1002/ana.23937 23686809 PMC3785076

[94] Brettschneider J Del Tredici K Irwin DJ Grossman M Robinson JL Toledo JB. Sequential distribution of pTDP-43 pathology in behavioral variant frontotemporal dementia (bvFTD). Acta Neuropathol. (2014) 127:423–39. 10.1007/s00401-013-1238-y 24407427 PMC3971993

[95] Brettschneider J Arai K Del Tredici K Toledo JB Robinson JL Lee EB. TDP-43 pathology and neuronal loss in amyotrophic lateral sclerosis spinal cord. Acta Neuropathol. (2014) 128:423–37. 10.1007/s00401-014-1299-6 24916269 PMC4384652

[96] Arai T Hasegawa M Akiyama H Ikeda K Nonaka T Mori H. TDP-43 is a component of ubiquitin-positive tau-negative inclusions in frontotemporal lobar degeneration and amyotrophic lateral sclerosis. Biochem Biophys Res Commun. (2006) 351:602–11. 10.1016/j.bbrc.2006.10.093 17084815

[97] Prasad A Bharathi V Sivalingam V Girdhar A Patel BK. Molecular mechanisms of TDP-43 misfolding and pathology in amyotrophic lateral sclerosis. Front Mol Neurosci. (2019) 12:25. 10.3389/fnmol.2019.00025 30837838 PMC6382748

[98] Mackenzie IRA Neumann M Baborie A Sampathu DM Du Plessis D Jaros E. A harmonized classification system for FTLD-TDP pathology. Acta Neuropathol. (2011) 122:111–3. 10.1007/s00401-011-0845-8 21644037 PMC3285143

[99] Forman MS Mackenzie IR Cairns NJ Swanson E Boyer PJ Drachman DA. Novel ubiquitin neuropathology in frontotemporal dementia with *Valosin-Containing Protein* gene mutations. J Neuropathol Exp Neurol. (2006) 65:571–81. 10.1097/00005072-200606000-00005 16783167

[100] Mackenzie IR Neumann M. Reappraisal of TDP-43 pathology in FTLD-U subtypes. Acta Neuropathol. (2017) 134:79–96. 10.1007/s00401-017-1716-8 28466142

[101] Lee EB Porta S Michael Baer G Xu Y Suh E Kwong LK. Expansion of the classification of FTLD-TDP: distinct pathology associated with rapidly progressive frontotemporal degeneration. Acta Neuropathol. (2017) 134:65–78. 10.1007/s00401-017-1679-9 28130640 PMC5521959

[102] Cushman M Johnson BS King OD Gitler AD Shorter J. Prion-like disorders: blurring the divide between transmissibility and infectivity. J Cell Sci. (2010) 123:1191–201. 10.1242/jcs.051672 20356930 PMC2848109

[103] Nonaka T Masuda-Suzukake M Arai T Hasegawa Y Akatsu H Obi T. Prion-like properties of pathological TDP-43 aggregates from diseased brains. Cell Rep. (2013) 4:124–34. 10.1016/j.celrep.2013.06.007 23831027

[104] Braak H Ludolph AC Neumann M Ravits J Del Tredici K. Pathological TDP-43 changes in Betz cells differ from those in bulbar and spinal α-motoneurons in sporadic amyotrophic lateral sclerosis. Acta Neuropathol. (2017) 133:79–90. 10.1007/s00401-016-1633-2 27757524 PMC5209403

[105] Maniecka Z Polymenidou M. From nucleation to widespread propagation: a prion-like concept for ALS. Virus Res. (2015) 207:94–105. 10.1016/j.virusres.2014.12.032 25656065

[106] Nana AL Sidhu M Gaus SE Hwang J-HL Li L Park Y. Neurons selectively targeted in frontotemporal dementia reveal early stage TDP-43 pathobiology. Acta Neuropathol. (2019) 137:27–46. 10.1007/s00401-018-1942-8 30511086 PMC6339592

[107] Prudlo J König J Schuster C Kasper E Büttner A Teipel S. TDP-43 pathology and cognition in ALS: a prospective clinicopathologic correlation study. Neurology. (2016) 87:1019–23. 10.1212/WNL.0000000000003062 27488596

[108] Cykowski MD Powell SZ Peterson LE Appel JW Rivera AL Takei H. Clinical significance of TDP-43 neuropathology in amyotrophic lateral sclerosis. J Neuropathol Exp Neurol. (2017) 76:402–13. 10.1093/jnen/nlx025 28521037 PMC5901081

[109] Henstridge CM Sideris DI Carroll E Rotariu S Salomonsson S Tzioras M. Synapse loss in the prefrontal cortex is associated with cognitive decline in amyotrophic lateral sclerosis. Acta Neuropathol. (2018) 135:213–26. 10.1007/s00401-017-1797-4 29273900 PMC5773656

[110] Starr A Sattler R. Synaptic dysfunction and altered excitability in C9ORF72 ALS/FTD. Brain Res. (2018) 1693:98–108. 10.1016/j.brainres.2018.02.011 29453960 PMC5997509

[111] Kovacs GG Alafuzoff I Al-Sarraj S Arzberger T Bogdanovic N Capellari S. Mixed brain pathologies in dementia: the brainnet Europe consortium experience. Dement Geriatr Cogn Disord. (2008) 26:343–50. 10.1159/000161560 18849605

[112] Rahimi J Kovacs GG. Prevalence of mixed pathologies in the aging brain. Alz Res Therapy. (2014) 6:82. 10.1186/s13195-014-0082-1 25419243 PMC4239398

[113] McCauley ME Baloh RH. Inflammation in ALS/FTD pathogenesis. Acta Neuropathol. (2019) 137:715–30. 10.1007/s00401-018-1933-9 30465257 PMC6482122

[114] Graves MC Fiala M Dinglasan LAV Liu NQ Sayre J Chiappelli F. Inflammation in amyotrophic lateral sclerosis spinal cord and brain is mediated by activated macrophages, mast cells and T cells. Amyotroph Lateral Scler Other Motor Neuron Disord. (2004) 5:213–9. 10.1080/14660820410020286 15799549

[115] Kawamata T Akiyama H Yamada T McGeer PL. Immunologic reactions in amyotrophic lateral sclerosis brain and spinal cord tissue. Am J Pathol. (1992) 140:691–707. 1347673 PMC1886170

[116] Engelhardt JI Tajti J Appel SH. Lymphocytic infiltrates in the spinal cord in amyotrophic lateral sclerosis. Arch Neurol. (1993) 50:30–6. 10.1001/archneur.1993.00540010026013 8093428

[117] Donnenfeld H Kascsak RJ Bartfeld H. Deposits of IgG and C3 in the spinal cord and motor cortex of ALS patients. J Neuroimmunol. (1984) 6:51–7. 10.1016/0165-5728(84)90042-0 6368581

[118] Sta M Sylva-Steenland RMR Casula M de Jong JMBV Troost D Aronica E. Innate and adaptive immunity in amyotrophic lateral sclerosis: evidence of complement activation. Neurobiol Dis. (2011) 42:211–20. 10.1016/j.nbd.2011.01.002 21220013

[119] Brettschneider J Toledo JB Van Deerlin VM Elman L McCluskey L Lee VM-Y. Microglial activation correlates with disease progression and upper motor neuron clinical symptoms in amyotrophic lateral sclerosis. PLoS ONE. (2012) 7:e39216. 10.1371/journal.pone.0039216 22720079 PMC3375234

[120] Brettschneider J Libon DJ Toledo JB Xie SX McCluskey L Elman L. Microglial activation and TDP-43 pathology correlate with executive dysfunction in amyotrophic lateral sclerosis. Acta Neuropathol. (2012) 123:395–407. 10.1007/s00401-011-0932-x 22210083 PMC3595560

[121] Paolicelli RC Jawaid A Henstridge CM Valeri A Merlini M Robinson JL. TDP-43 depletion in microglia promotes amyloid clearance but also induces synapse loss. Neuron. (2017) 95:297–308.e6. 10.1016/j.neuron.2017.05.037 28669544 PMC5519492

[122] Vahsen BF Gray E Thompson AG Ansorge O Anthony DC Cowley SA. Non-neuronal cells in amyotrophic lateral sclerosis — from pathogenesis to biomarkers. Nat Rev Neurol. (2021) 17:333–48. 10.1038/s41582-021-00487-8 33927394

[123] Nagy D Kato T Kushner PD. Reactive astrocytes are widespread in the cortical gray matter of amyotrophic lateral sclerosis. J Neurosci Res. (1994) 38:336–47. 10.1002/jnr.490380312 7523689

[124] Altmann A Cash DM Bocchetta M Heller C Reynolds R Moore K. Analysis of brain atrophy and local gene expression in genetic frontotemporal dementia. Brain Commun. (2020) 2:fcaa122. 10.1101/2019.12.11.872143 33210084 PMC7667525

[125] Tam OH Rozhkov NV Shaw R Kim D Hubbard I Fennessey S. Postmortem cortex samples identify distinct molecular subtypes of ALS: retrotransposon activation, oxidative stress, and activated glia. Cell Rep. (2019) 29:1164–77.e5. 10.1016/j.celrep.2019.09.066 31665631 PMC6866666

[126] Nolan M Scott C Gamarallage MP Lunn D Carpenter K McDonough E. Quantitative patterns of motor cortex proteinopathy across ALS genotypes. Acta Neuropathol Commun. (2020) 8:98. 10.1186/s40478-020-00961-2 32616036 PMC7331195

[127] Mackenzie IR Neumann M. Subcortical TDP-43 pathology patterns validate cortical FTLD-TDP subtypes and demonstrate unique aspects of C9orf72 mutation cases. Acta Neuropathol. (2020) 139:83–98. 10.1007/s00401-019-02070-4 31501924

[128] Mackenzie IRA Frick P Neumann M. The neuropathology associated with repeat expansions in the C9ORF72 gene. Acta Neuropathol. (2014) 127:347–57. 10.1007/s00401-013-1232-4 24356984

[129] Lleó A Cavedo E Parnetti L Vanderstichele H Herukka SK Andreasen N. Cerebrospinal fluid biomarkers in trials for Alzheimer and Parkinson diseases. Nat Rev Neurol. (2015) 11:41–55. 10.1038/nrneurol.2014.232 25511894

[130] Zetterberg H. Applying fluid biomarkers to Alzheimer's disease. Am J Physiol Cell Physiol. (2017) 313:C3–10. 10.1152/ajpcell.00007.2017 28424166 PMC5538797

[131] Agah E Saleh F Sanjari Moghaddam H Saghazadeh A Tafakhori A Rezaei N. CSF and blood biomarkers in amyotrophic lateral sclerosis: protocol for a systematic review and meta-analysis. Syst Rev. (2018) 7:237. 10.1186/s13643-018-0913-4 30572951 PMC6300914

[132] Yuan A Rao MV Veeranna null Nixon RA. Neurofilaments and neurofilament proteins in health and disease. Cold Spring Harb Perspect Biol. (2017) 9:a018309. 10.1101/cshperspect.a018309 28373358 PMC5378049

[133] Bridel C van Wieringen WN Zetterberg H Tijms BM Teunissen CE the NFL Group. Diagnostic value of cerebrospinal fluid neurofilament light protein in neurology: a systematic review and meta-analysis. JAMA Neurol. (2019) 76:1035–48. 10.1001/jamaneurol.2019.1534 31206160 PMC6580449

[134] Delaby C Alcolea D Carmona-Iragui M Illán-Gala I Morenas-Rodríguez E Barroeta I. Differential levels of neurofilament light protein in cerebrospinal fluid in patients with a wide range of neurodegenerative disorders. Sci Rep. (2020) 10:9161. 10.1038/s41598-020-66090-x 32514050 PMC7280194

[135] Steinacker P Feneberg E Weishaupt J Brettschneider J Tumani H Andersen PM. Neurofilaments in the diagnosis of motoneuron diseases: a prospective study on 455 patients. J Neurol Neurosurg Psychiatry. (2016) 87:12–20. 10.1136/jnnp-2015-311387 26296871

[136] Feneberg E Oeckl P Steinacker P Verde F Barro C Van Damme P. Multicenter evaluation of neurofilaments in early symptom onset amyotrophic lateral sclerosis. Neurology. (2018) 90:e22–30. 10.1212/WNL.0000000000004761 29212830

[137] Verde F Steinacker P Weishaupt JH Kassubek J Oeckl P Halbgebauer S. Neurofilament light chain in serum for the diagnosis of amyotrophic lateral sclerosis. J Neurol Neurosurg Psychiatry. (2019) 90:157–64. 10.1136/jnnp-2018-318704 30309882

[138] Scherling CS Hall T Berisha F Klepac K Karydas A Coppola G. Cerebrospinal fluid neurofilament concentration reflects disease severity in frontotemporal degeneration: neurofilament in FTD. Ann Neurol. (2014) 75:116–26. 10.1002/ana.24052 24242746 PMC4020786

[139] Menke RAL Gray E Lu C-H Kuhle J Talbot K Malaspina A. CSF neurofilament light chain reflects corticospinal tract degeneration in ALS. Ann Clin Transl Neurol. (2015) 2:748–55. 10.1002/acn3.212 26273687 PMC4531057

[140] Alcolea D Vilaplana E Suárez-Calvet M Illán-Gala I Blesa R Clarimón J. CSF sAPPβ, YKL-40, and neurofilament light in frontotemporal lobar degeneration. Neurology. (2017) 89:178–88. 10.1212/WNL.0000000000004088 28592456

[141] Forgrave LM Ma M Best JR DeMarco ML. The diagnostic performance of neurofilament light chain in CSF and blood for Alzheimer's disease, frontotemporal dementia, and amyotrophic lateral sclerosis: a systematic review and meta-analysis. Alzheimes Demen Diag Assess Dis Monit. (2019) 11:730–43. 10.1016/j.dadm.2019.08.009 31909174 PMC6939029

[142] Steinacker P Anderl-Straub S Diehl-Schmid J Semler E Uttner I von Arnim CAF. Serum neurofilament light chain in behavioral variant frontotemporal dementia. Neurology. (2018) 91:e1390–401. 10.1212/WNL.0000000000006318 30209235

[143] Gaiani A Martinelli I Bello L Querin G Puthenparampil M Ruggero S. Diagnostic and prognostic biomarkers in amyotrophic lateral sclerosis: neurofilament light chain levels in definite subtypes of disease. JAMA Neurol. (2017) 74:525. 10.1001/jamaneurol.2016.5398 28264096 PMC5822207

[144] Landqvist Waldö M Frizell Santillo A Passant U Zetterberg H Rosengren L Nilsson C. Cerebrospinal fluid neurofilament light chain protein levels in subtypes of frontotemporal dementia. BMC Neurol. (2013) 13:54. 10.1186/1471-2377-13-54 23718879 PMC3671150

[145] Meeter LHH Gendron TF Sias AC Jiskoot LC Russo SP Donker Kaat L. Poly(GP), neurofilament and grey matter deficits in *C9orf72* expansion carriers. Ann Clin Transl Neurol. (2018) 5:583–97. 10.1002/acn3.559 29761121 PMC5945959

[146] Rohrer JD Woollacott IOC Dick KM Brotherhood E Gordon E Fellows A. Serum neurofilament light chain protein is a measure of disease intensity in frontotemporal dementia. Neurology. (2016) 87:1329–36. 10.1212/WNL.0000000000003154 27581216 PMC5047041

[147] Skillbäck T Farahmand B Bartlett JW Rosén C Mattsson N Nägga K. CSF neurofilament light differs in neurodegenerative diseases and predicts severity and survival. Neurology. (2014) 83:1945–53. 10.1212/WNL.0000000000001015 25339208

[148] Wilke C Preische O Deuschle C Roeben B Apel A Barro C. Neurofilament light chain in FTD is elevated not only in cerebrospinal fluid, but also in serum. J Neurol Neurosurg Psychiatry. (2016) 87:1270–2. 10.1136/jnnp-2015-312972 27188986

[149] Lu C-H Macdonald-Wallis C Gray E Pearce N Petzold A Norgren N. Neurofilament light chain: a prognostic biomarker in amyotrophic lateral sclerosis. Neurology. (2015) 84:2247–57. 10.1212/WNL.0000000000001642 25934855 PMC4456658

[150] Rojas JC Karydas A Bang J Tsai RM Blennow K Liman V. Plasma neurofilament light chain predicts progression in progressive supranuclear palsy. Ann Clin Transl Neurol. (2016) 3:216–25. 10.1002/acn3.290 27042681 PMC4774256

[151] Bright F Werry EL Dobson-Stone C Piguet O Ittner LM Halliday GM. Neuroinflammation in frontotemporal dementia. Nat Rev Neurol. (2019) 15:540–55. 10.1038/s41582-019-0231-z 31324897

[152] Philips T Robberecht W. Neuroinflammation in amyotrophic lateral sclerosis: role of glial activation in motor neuron disease. Lancet Neurol. (2011) 10:253–63. 10.1016/S1474-4422(11)70015-1 21349440

[153] Thompson AG Turner MR. Untangling neuroinflammation in amyotrophic lateral sclerosis. J Neurol Neurosurg Psychiatry. (2019) 90:1303–4. 10.1136/jnnp-2019-321242 31296587

[154] Ashton NJ Janelidze S Al Khleifat A Leuzy A van der Ende EL Karikari TK. A multicentre validation study of the diagnostic value of plasma neurofilament light. Nat Commun. (2021) 12:3400. 10.1038/s41467-021-23620-z 34099648 PMC8185001

[155] Halbgebauer S Steinacker P Verde F Weishaupt J Oeckl P von Arnim C. Comparison of CSF and serum neurofilament light and heavy chain as differential diagnostic biomarkers for ALS. J Neurol Neurosurg Psychiatry. (2022) 93:68–74. 10.1136/jnnp-2021-327129 34417339

[156] Verde F Otto M Silani V. Neurofilament light chain as biomarker for amyotrophic lateral sclerosis and frontotemporal dementia. Front Neurosci. (2021) 15:679199. 10.3389/fnins.2021.679199 34234641 PMC8255624

[157] Ahmed RM Phan K Highton-Williamson E Strikwerda-Brown C Caga J Ramsey E. Eating peptides: biomarkers of neurodegeneration in amyotrophic lateral sclerosis and frontotemporal dementia. Ann Clin Transl Neurol. (2019) 6:486–95. 10.1002/acn3.721 30911572 PMC6414477

[158] Illán-Gala I Montal V Pegueroles J Vilaplana E Alcolea D Dols-Icardo O. Cortical microstructure in the amyotrophic lateral sclerosis–frontotemporal dementia continuum. Neurology. (2020) 95:e2565–76. 10.1212/WNL.0000000000010727 32913016 PMC7682829

[159] Borroni B Benussi A Archetti S Galimberti D Parnetti L Nacmias B. Csf p-tau _181_ /tau ratio as biomarker for TDP pathology in frontotemporal dementia. Amyotrophic Later Scler Frontotemporal Degener. (2015) 16:86–91. 10.3109/21678421.2014.971812 25352065

[160] Grossman M Elman L McCluskey L McMillan CT Boller A Powers J. Phosphorylated tau as a candidate biomarker for amyotrophic lateral sclerosis. JAMA Neurol. (2014) 71:442. 10.1001/jamaneurol.2013.6064 24492862 PMC3989393

[161] Hu WT Watts K Grossman M Glass J Lah JJ Hales C. Reduced CSF p-Tau181 to tau ratio is a biomarker for FTLD-TDP. Neurology. (2013) 81:1945–52. 10.1212/01.wnl.0000436625.63650.27 24174584 PMC3843382

[162] Meeter LHH Vijverberg EG Del Campo M Rozemuller AJM Donker Kaat L de Jong FJ. Clinical value of neurofilament and phospho-tau/tau ratio in the frontotemporal dementia spectrum. Neurology. (2018) 90:e1231–9. 10.1212/WNL.0000000000005261 29514947 PMC5890612

[163] Kuiperij HB Versleijen AAM Beenes M Verwey NA Benussi L Paterlini A. Tau rather than TDP-43 proteins are potential cerebrospinal fluid biomarkers for frontotemporal lobar degeneration subtypes: a pilot study. J Alzheimers Dis. (2017) 55:585–95. 10.3233/JAD-160386 27662293

[164] Pijnenburg YAL Verwey NA van der Flier WM Scheltens P Teunissen CE. Discriminative and prognostic potential of cerebrospinal fluid phosphoTau/tau ratio and neurofilaments for frontotemporal dementia subtypes. Alzheimers Dement. (2015) 1:505–12. 10.1016/j.dadm.2015.11.001 27239528 PMC4879490

[165] Lleó A Irwin DJ Illán-Gala I McMillan CT Wolk DA Lee EB. A 2-step cerebrospinal algorithm for the selection of frontotemporal lobar degeneration subtypes. JAMA Neurol. (2018) 75:738–45. 10.1001/jamaneurol.2018.0118 29554190 PMC5885205

[166] Müller UC Deller T Korte M. Not just amyloid: physiological functions of the amyloid precursor protein family. Nat Rev Neurosci. (2017) 18:281–98. 10.1038/nrn.2017.29 28360418

[167] Ghidoni R Benussi L Paterlini A Albertini V Binetti G Emanuele E. Cerebrospinal fluid biomarkers for Alzheimer's disease: the present and the future. Neurodegener Dis. (2011) 8:413–20. 10.1159/000327756 21709402

[168] Nhan HS Chiang K Koo EH. The multifaceted nature of amyloid precursor protein and its proteolytic fragments: friends and foes. Acta Neuropathol. (2015) 129:1–19. 10.1007/s00401-014-1347-2 25287911 PMC4282969

[169] Portelius E Price E Brinkmalm G Stiteler M Olsson M Persson R. A novel pathway for amyloid precursor protein processing. Neurobiol Aging. (2011) 32:1090–8. 10.1016/j.neurobiolaging.2009.06.002 19604603

[170] Cirrito JR Yamada KA Finn MB Sloviter RS Bales KR May PC. Synaptic activity regulates interstitial fluid amyloid-beta levels in vivo. Neuron. (2005) 48:913–22. 10.1016/j.neuron.2005.10.028 16364896

[171] Illán-Gala I Pegueroles J Montal V Alcolea D Vilaplana E Bejanin A. APP-derived peptides reflect neurodegeneration in frontotemporal dementia. Ann Clin Transl Neurol. (2019) 6:2518–30. 10.1002/acn3.50948 31789459 PMC6917306

[172] Mucke L Selkoe DJ. Neurotoxicity of amyloid β-protein: synaptic and network dysfunction. Cold Spring Harb Perspect Med. (2012) 2:a006338. 10.1101/cshperspect.a006338 22762015 PMC3385944

[173] Alcolea D Irwin DJ Illán-Gala I Muñoz L Clarimón J McMillan CT. Elevated YKL-40 and low sAPPβ:YKL-40 ratio in antemortem cerebrospinal fluid of patients with pathologically confirmed FTLD. J Neurol Neurosurg Psychiatry. (2019) 90:180–6. 10.1136/jnnp-2018-318993 30297518 PMC6351153

[174] Illán-Gala I Alcolea D Montal V Dols-Icardo O Muñoz L de Luna N. CSF sAPPβ, YKL-40, and NfL along the ALS-FTD spectrum. Neurology. (2018) 91:e1619–28. 10.1212/WNL.0000000000006383 30291183

[175] Perneczky R Guo L-H Kagerbauer SM Werle L Kurz A Martin J. Soluble amyloid precursor protein β as blood-based biomarker of Alzheimer's disease. Transl Psychiatry. (2013) 3:e227. 10.1038/tp.2013.11 23423136 PMC3591004

[176] Portelius E Olsson B Höglund K Cullen NC Kvartsberg H Andreasson U. Cerebrospinal fluid neurogranin concentration in neurodegeneration: relation to clinical phenotypes and neuropathology. Acta Neuropathol. (2018) 136:363–76. 10.1007/s00401-018-1851-x 29700597 PMC6096740

[177] Collins MA An J Hood BL Conrads TP Bowser RP. Label-Free LC–MS/MS proteomic analysis of cerebrospinal fluid identifies protein/pathway alterations and candidate biomarkers for amyotrophic lateral sclerosis. J Proteome Res. (2015) 14:4486–501. 10.1021/acs.jproteome.5b00804 26401960 PMC5592736

[178] Belbin O Xiao M-F Xu D Carmona-Iragui M Pegueroles J Benejam B. Cerebrospinal fluid profile of NPTX2 supports role of Alzheimer's disease-related inhibitory circuit dysfunction in adults with down syndrome. Mol Neurodegener. (2020) 15:46. 10.1186/s13024-020-00398-0 32807227 PMC7433053

[179] Lleó A Núñez-Llaves R Alcolea D Chiva C Balateu-Paños D Colom-Cadena M. Changes in synaptic proteins precede neurodegeneration markers in preclinical Alzheimer's disease cerebrospinal fluid. Mol Cell Proteomics. (2019) 18:546–60. 10.1074/mcp.RA118.001290 30606734 PMC6398205

[180] Sjögren M Folkesson S Blennow K Tarkowski E. Increased intrathecal inflammatory activity in frontotemporal dementia: pathophysiological implications. J Neurol Neurosurg Psychiatry. (2004) 75:1107–11. 10.1136/jnnp.2003.019422 15258209 PMC1739153

[181] Beers DR Appel SH. Immune dysregulation in amyotrophic lateral sclerosis: mechanisms and emerging therapies. Lancet Neurol. (2019) 18:211–20. 10.1016/S1474-4422(18)30394-6 30663610

[182] Hu Y Cao C Qin X-Y Yu Y Yuan J Zhao Y. Increased peripheral blood inflammatory cytokine levels in amyotrophic lateral sclerosis: a meta-analysis study. Sci Rep. (2017) 7:9094. 10.1038/s41598-017-09097-1 28831083 PMC5567306

[183] Rentzos M Rombos A Nikolaou C Zoga M Zouvelou V Dimitrakopoulos A. Interleukin-17 and interleukin-23 are elevated in serum and cerebrospinal fluid of patients with ALS: a reflection of Th17 cells activation?: circulating IL-17 and IL-23 in ALS. Acta Neurol Scand. (2010) 122:425–9. 10.1111/j.1600-0404.2010.01333.x 20219021

[184] Boström G Freyhult E Virhammar J Alcolea D Tumani H Otto M. Different inflammatory signatures in alzheimer's disease and frontotemporal dementia cerebrospinal fluid. J Alzheimers Dis. (2021) 81:629–40. 10.3233/JAD-201565 33814444 PMC8203220

[185] Appel SH Beers DR Zhao W. Amyotrophic lateral sclerosis is a systemic disease: peripheral contributions to inflammation-mediated neurodegeneration. Curr Opin Neurol. (2021) 34:765–72. 10.1097/WCO.0000000000000983 34402459

[186] Mitchell RM Freeman WM Randazzo WT Stephens HE Beard JL Simmons Z. A CSF biomarker panel for identification of patients with amyotrophic lateral sclerosis. Neurology. (2009) 72:14–9. 10.1212/01.wnl.0000333251.36681.a5 18987350

[187] Thompson AG Gray E Thézénas M-L Charles PD Evetts S Hu MT. Cerebrospinal fluid macrophage biomarkers in amyotrophic lateral sclerosis: CSF macrophage biomarkers in ALS. Ann Neurol. (2018) 83:258–68. 10.1002/ana.25143 29331073

[188] Bonneh-Barkay D Wang G Starkey A Hamilton RL Wiley CA. *In vivo* CHI3L1 (YKL-40) expression in astrocytes in acute and chronic neurological diseases. J Neuroinflammation. (2010) 7:34. 10.1186/1742-2094-7-34 20540736 PMC2892443

[189] Benussi A Ashton NJ Karikari TK Gazzina S Premi E Benussi L. Serum glial fibrillary acidic protein (GFAP) is a marker of disease severity in frontotemporal lobar degeneration. J Alzheimers Dis. (2020) 77:1129–41. 10.3233/JAD-200608 32804092

[190] Oeckl P Weydt P Steinacker P Anderl-Straub S Nordin F Volk AE. Different neuroinflammatory profile in amyotrophic lateral sclerosis and frontotemporal dementia is linked to the clinical phase. J Neurol Neurosurg Psychiatry. (2019) 90:4–10. 10.1136/jnnp-2018-318868 30224549

[191] Cady J Koval ED Benitez BA Zaidman C Jockel-Balsarotti J Allred P. TREM2 variant p.R47H as a risk factor for sporadic amyotrophic lateral sclerosis. JAMA Neurol. (2014) 71:449–53. 10.1001/jamaneurol.2013.6237 24535663 PMC4087113

[192] Lill CM Rengmark A Pihlstrøm L Fogh I Shatunov A Sleiman PM. The role of TREM2 R47H as a risk factor for Alzheimer's disease, frontotemporal lobar degeneration, amyotrophic lateral sclerosis, and Parkinson's disease. Alzheimers Dement. (2015) 11:1407–16. 10.1016/j.jalz.2014.12.009 25936935 PMC4627856

[193] Siokas V Aloizou A-M Liampas I Tsouris Z Mentis A-FA Nasios G. Lack of association between TREM2 rs75932628 variant and amyotrophic lateral sclerosis. Mol Biol Rep. (2021) 48:2601–10. 10.1007/s11033-021-06312-1 33826063

[194] Woollacott IOC Nicholas JM Heslegrave A Heller C Foiani MS Dick KM. Cerebrospinal fluid soluble TREM2 levels in frontotemporal dementia differ by genetic and pathological subgroup. Alzheimers Res Ther. (2018) 10:79. 10.1186/s13195-018-0405-8 30111356 PMC6094471

[195] Ganesalingam J An J Shaw CE Shaw G Lacomis D Bowser R. Combination of neurofilament heavy chain and complement C3 as CSF biomarkers for ALS. J Neurochem. (2011) 117:528–37. 10.1111/j.1471-4159.2011.07224.x 21418221 PMC3076545

[196] Goldknopf IL Sheta EA Bryson J Folsom B Wilson C Duty J. Complement C3c and related protein biomarkers in amyotrophic lateral sclerosis and Parkinson's disease. Biochem Biophys Res Commun. (2006) 342:1034–9. 10.1016/j.bbrc.2006.02.051 16516157

[197] Majumder V Gregory JM Barria MA Green A Pal S. TDP-43 as a potential biomarker for amyotrophic lateral sclerosis: a systematic review and meta-analysis. BMC Neurol. (2018) 18:90. 10.1186/s12883-018-1091-7 29954341 PMC6027783

[198] Foulds PG Davidson Y Mishra M Hobson DJ Humphreys KM Taylor M. Plasma phosphorylated-TDP-43 protein levels correlate with brain pathology in frontotemporal lobar degeneration. Acta Neuropathol. (2009) 118:647–58. 10.1007/s00401-009-0594-0 19823856 PMC2809136

[199] Junttila A Kuvaja M Hartikainen P Siloaho M Helisalmi S Moilanen V. Cerebrospinal fluid TDP-43 in frontotemporal lobar degeneration and amyotrophic lateral sclerosis patients with and without the C9ORF72 hexanucleotide expansion. Dement Geriatr Cogn Disord Extra. (2016) 6:142–9. 10.1159/000444788 27195002 PMC4868946

[200] Feneberg E Gray E Ansorge O Talbot K Turner MR. Towards a TDP-43-based biomarker for ALS and FTLD. Mol Neurobiol. (2018) 55:7789–801. 10.1007/s12035-018-0947-6 29460270 PMC6132775

[201] Kasai T Kojima Y Ohmichi T Tatebe H Tsuji Y Noto Y. Combined use of CSF NfL and CSF TDP-43 improves diagnostic performance in ALS. Ann Clin Transl Neurol. (2019) 6:2489–502. 10.1002/acn3.50943 31742901 PMC6917342

[202] Beyer L Günther R Koch JC Klebe S Hagenacker T Lingor P. TDP-43 as structure-based biomarker in amyotrophic lateral sclerosis. Ann Clin Transl Neurol. (2021) 8:271–7. 10.1002/acn3.51256 33263951 PMC7818221

[203] Scialò C Tran TH Salzano G Novi G Caponnetto C Chiò A. TDP-43 real-time quaking induced conversion reaction optimization and detection of seeding activity in CSF of amyotrophic lateral sclerosis and frontotemporal dementia patients. Brain Commun. (2020) 2:fcaa142. 10.1093/braincomms/fcaa142 33094285 PMC7566418

[204] Prudencio M Humphrey J Pickles S Brown A-L Hill SE Kachergus JM. Truncated stathmin-2 is a marker of TDP-43 pathology in frontotemporal dementia. J Clin Invest. (2020) 130:6080–92. 10.1172/JCI139741 32790644 PMC7598060

[205] Lehmer C Oeckl P Weishaupt JH Volk AE Diehl-Schmid J Schroeter ML. Poly- GP in cerebrospinal fluid links *C9orf72*-associated dipeptide repeat expression to the asymptomatic phase of ALS/FTD. EMBO Mol Med. (2017) 9:859–68. 10.15252/emmm.201607486 28408402 PMC5494528

[206] Kassubek J Pagani M. Imaging in amyotrophic lateral sclerosis: MRI and PET. Curr Opin Neurol. (2019) 32:740–6. 10.1097/WCO.0000000000000728 31335337

[207] Kalra S Müller H-P Ishaque A Zinman L Korngut L Genge A. A prospective harmonized multicenter DTI study of cerebral white matter degeneration in ALS. Neurology. (2020) 95:e943–52. 10.1212/WNL.0000000000010235 32646955 PMC7668555

[208] Agosta F Spinelli EG Filippi M. Neuroimaging in amyotrophic lateral sclerosis: current and emerging uses. Expert Rev Neurother. (2018) 18:395–406. 10.1080/14737175.2018.1463160 29630421

[209] Chen Z Ma L. Grey matter volume changes over the whole brain in amyotrophic lateral sclerosis: A voxel-wise meta-analysis of voxel based morphometry studies. Amyotroph Lateral Scler. (2010) 11:549–54. 10.3109/17482968.2010.516265 20929296

[210] Shen D Cui L Fang J Cui B Li D Tai H. Voxel-Wise meta-analysis of gray matter changes in amyotrophic lateral sclerosis. Front Aging Neurosci. (2016) 8:64. 10.3389/fnagi.2016.00064 27065078 PMC4811926

[211] Consonni M Dalla Bella E Contarino VE Bersano E Lauria G. Cortical thinning trajectories across disease stages and cognitive impairment in amyotrophic lateral sclerosis. Cortex. (2020) 131:284–94. 10.1016/j.cortex.2020.07.007 32811660

[212] Consonni M Contarino VE Catricalà E Dalla Bella E Pensato V Gellera C. Cortical markers of cognitive syndromes in amyotrophic lateral sclerosis. NeuroImage Clin. (2018) 19:675–82. 10.1016/j.nicl.2018.05.020 30023173 PMC6046611

[213] Bede P Elamin M Byrne S McLaughlin RL Kenna K Vajda A. Basal ganglia involvement in amyotrophic lateral sclerosis. Neurology. (2013) 81:2107–15. 10.1212/01.wnl.0000437313.80913.2c 24212388

[214] Westeneng H-J Walhout R Straathof M Schmidt R Hendrikse J Veldink JH. Widespread structural brain involvement in ALS is not limited to the C9orf72 repeat expansion. J Neurol Neurosurg Psychiatry. (2016) 87:1354–60. 10.1136/jnnp-2016-313959 27756805 PMC5136726

[215] Dadar M Manera AL Zinman L Korngut L Genge A Graham SJ. Cerebral atrophy in amyotrophic lateral sclerosis parallels the pathological distribution of TDP43. Brain Commun. (2020) 2:fcaa061. 10.1093/braincomms/fcaa061 33543125 PMC7846188

[216] Illán-Gala I Montal V Borrego-Écija S Vilaplana E Pegueroles J Alcolea D. Cortical microstructure in the behavioural variant of frontotemporal dementia: looking beyond atrophy. Brain. (2019) 142:1121–33. 10.1093/brain/awz031 30906945 PMC6439330

[217] Le Bihan D. Looking into the functional architecture of the brain with diffusion MRI. Nat Rev Neurosci. (2003) 4:469–80. 10.1038/nrn1119 12778119

[218] Weston PSJ Simpson IJA Ryan NS Ourselin S Fox NC. Diffusion imaging changes in grey matter in Alzheimer's disease: a potential marker of early neurodegeneration. Alzheimers Res Ther. (2015) 7:47. 10.1186/s13195-015-0132-3 26136857 PMC4487800

[219] Vilaplana E Rodriguez-Vieitez E Ferreira D Montal V Almkvist O Wall A. Cortical microstructural correlates of astrocytosis in autosomal-dominant Alzheimer disease. Neurology. (2020) 94:e2026–36. 10.1212/WNL.0000000000009405 32291295 PMC7282881

[220] Montal V Vilaplana E Alcolea D Pegueroles J Pasternak O González-Ortiz S. Cortical microstructural changes along the Alzheimer's disease continuum. Alzheimers Dement. (2018) 14:340–51. 10.1016/j.jalz.2017.09.013 29080407

[221] Montal V Vilaplana E Pegueroles J Bejanin A Alcolea D Carmona-Iragui M. Biphasic cortical macro- and microstructural changes in autosomal dominant Alzheimer's disease. Alzheimers Dement. (2021) 17:618–28. 10.1002/alz.12224 33196147 PMC8043974

[222] van der Burgh HK Westeneng H-J Walhout R van Veenhuijzen K Tan HHG Meier JM. Multimodal longitudinal study of structural brain involvement in amyotrophic lateral sclerosis. Neurology. (2020) 94:e2592–604. 10.1212/WNL.0000000000009498 32414878 PMC7455328

[223] Agosta F Ferraro PM Riva N Spinelli EG Chiò A Canu E. Structural brain correlates of cognitive and behavioral impairment in MND. Hum Brain Mapp. (2016) 37:1614–26. 10.1002/hbm.23124 26833930 PMC6867462

[224] Trojsi F Caiazzo G Siciliano M Femiano C Passaniti C Russo A. Microstructural correlates of edinburgh cognitive and behavioural ALS screen (ECAS) changes in amyotrophic lateral sclerosis. Psychiatry Res Neuroimag. (2019) 288:67–75. 10.1016/j.pscychresns.2019.04.001 30987770

[225] Menke RAL Körner S Filippini N Douaud G Knight S Talbot K. Widespread grey matter pathology dominates the longitudinal cerebral MRI and clinical landscape of amyotrophic lateral sclerosis. Brain. (2014) 137 (Pt. 9):2546–55. 10.1093/brain/awu162 24951638 PMC4132644

[226] Trojsi F Esposito F de Stefano M Buonanno D Conforti FL Corbo D. Functional overlap and divergence between ALS and bvFTD. Neurobiol Aging. (2015) 36:413–23. 10.1016/j.neurobiolaging.2014.06.025 25063233

[227] Agosta F Canu E Inuggi A Chiò A Riva N Silani V. Resting state functional connectivity alterations in primary lateral sclerosis. Neurobiol Aging. (2014) 35:916–25. 10.1016/j.neurobiolaging.2013.09.041 24211007

[228] Menke RAL Proudfoot M Wuu J Andersen PM Talbot K Benatar M. Increased functional connectivity common to symptomatic amyotrophic lateral sclerosis and those at genetic risk. J Neurol Neurosurg Psychiatry. (2016) 87:580–8. 10.1136/jnnp-2015-311945 26733601 PMC4893149

[229] Basaia S Agosta F Cividini C Trojsi F Riva N Spinelli EG. Structural and functional brain connectome in motor neuron diseases: a multicenter MRI study. Neurology. (2020) 95:e2552–64. 10.1212/WNL.0000000000010731 32913015 PMC7682834

[230] Kalra S. Magnetic resonance spectroscopy in ALS. Front Neurol. (2019) 10:482. 10.3389/fneur.2019.00482 31133975 PMC6524558

[231] Ernst T Chang L Melchor R Mehringer CM. Frontotemporal dementia and early Alzheimer disease: differentiation with frontal lobe H-1 MR spectroscopy. Radiology. (1997) 203:829–36. 10.1148/radiology.203.3.9169712 9169712

[232] Pohl C Block W Träber F Schmidt S Pels H Grothe C. Proton magnetic resonance spectroscopy and transcranial magnetic stimulation for the detection of upper motor neuron degeneration in ALS patients. J Neurol Sci. (2001) 190:21–7. 10.1016/S0022-510X(01)00568-8 11574102

[233] Kalra S Hanstock CC Martin WRW Allen PS Johnston WS. Detection of cerebral degeneration in amyotrophic lateral sclerosis using high-field magnetic resonance spectroscopy. Arch Neurol. (2006) 63:1144. 10.1001/archneur.63.8.1144 16908742

[234] Kalra S Vitale A Cashman NR Genge A Arnold DL. Cerebral degeneration predicts survival in amyotrophic lateral sclerosis. J Neurol Neurosurg Psychiatry. (2006) 77:1253–5. 10.1136/jnnp.2006.090696 16835288 PMC2077382

[235] Pyra T Hui B Hanstock C Concha L Wong JCT Beaulieu C. Combined structural and neurochemical evaluation of the corticospinal tract in amyotrophic lateral sclerosis. Amyotroph Lateral Scler. (2010) 11:157–65. 10.3109/17482960902756473 19242831

[236] Atassi N Xu M Triantafyllou C Keil B Lawson R Cernasov P. Ultra high-field (7tesla) magnetic resonance spectroscopy in amyotrophic lateral sclerosis. PLoS ONE. (2017) 12:e0177680. 10.1371/journal.pone.0177680 28498852 PMC5428977

[237] Liu C Jiang R Yi X Zhu W Bu B. Role of diffusion tensor imaging or magnetic resonance spectroscopy in the diagnosis and disability assessment of amyotrophic lateral sclerosis. J Neurol Sci. (2015) 348:206–10. 10.1016/j.jns.2014.12.004 25524526

[238] Chen Q Boeve BF Tosakulwong N Lesnick T Brushaber D Dheel C. Frontal lobe 1H MR spectroscopy in asymptomatic and symptomatic MAPT mutation carriers. Neurology. (2019) 93:e758–65. 10.1212/WNL.0000000000007961 31315971 PMC6711662

[239] Chen Q Boeve BF Tosakulwong N Lesnick T Brushaber D Dheel C. Brain MR spectroscopy changes precede frontotemporal lobar degeneration phenoconversion in mapt mutation carriers. J Neuroimaging. (2019) 29:624–9. 10.1111/jon.12642 31173437 PMC6731148

[240] Chételat G Arbizu J Barthel H Garibotto V Law I Morbelli S. Amyloid-PET and 18F-FDG-PET in the diagnostic investigation of Alzheimer's disease and other dementias. Lancet Neurol. (2020) 19:951–62. 10.1016/S1474-4422(20)30314-8 33098804

[241] Teipel S Drzezga A Grothe MJ Barthel H Chételat G Schuff N. Multimodal imaging in Alzheimer's disease: validity and usefulness for early detection. Lancet Neurol. (2015) 14:1037–53. 10.1016/S1474-4422(15)00093-9 26318837

[242] Zimmer ER Parent MJ Souza DG Leuzy A Lecrux C Kim H-I. [18F]FDG PET signal is driven by astroglial glutamate transport. Nat Neurosci. (2017) 20:393–5. 10.1038/nn.4492 28135241 PMC5378483

[243] Canosa A Moglia C Manera U Vasta R Torrieri MC Arena V. Metabolic brain changes across different levels of cognitive impairment in ALS: a 18F-FDG-PET study. J Neurol Neurosurg Psychiatry. (2020). 10.1136/jnnp-2020-323876. [Epub ahead of print]. 33229451

[244] Canosa A Pagani M Cistaro A Montuschi A Iazzolino B Fania P. 18F-FDG-PET correlates of cognitive impairment in ALS. Neurology. (2016) 86:44–9. 10.1212/WNL.0000000000002242 26590270

[245] Pagani M Chio A Valentini MC Oberg J Nobili F Calvo A. Functional pattern of brain FDG-PET in amyotrophic lateral sclerosis. Neurology. (2014) 83:1067–74. 10.1212/WNL.0000000000000792 25122207

[246] Van Laere K Vanhee A Verschueren J De Coster L Driesen A Dupont P. Value of ^18^ fluorodeoxyglucose–positron-emission tomography in amyotrophic lateral sclerosis: a prospective study. JAMA Neurol. (2014) 71:553. 10.1001/jamaneurol.2014.62 24615479

[247] Turner MR Cagnin A Turkheimer FE Miller CCJ Shaw CE Brooks DJ. Evidence of widespread cerebral microglial activation in amyotrophic lateral sclerosis: an [11C](R)-PK11195 positron emission tomography study. Neurobiol Dis. (2004) 15:601–9. 10.1016/j.nbd.2003.12.012 15056468

[248] Alshikho MJ Zürcher NR Loggia ML Cernasov P Chonde DB Izquierdo Garcia D. Glial activation colocalizes with structural abnormalities in amyotrophic lateral sclerosis. Neurology. (2016) 87:2554–61. 10.1212/WNL.0000000000003427 27837005 PMC5207001

[249] Cagnin A Rossor M Sampson EL MacKinnon T Banati RB. In vivo detection of microglial activation in frontotemporal dementia. Ann Neurol. (2004) 56:894–7. 10.1002/ana.20332 15562429

[250] Corcia P Tauber C Vercoullie J Arlicot N Prunier C Praline J. Molecular imaging of microglial activation in amyotrophic lateral sclerosis. PLoS ONE. (2012) 7:e52941. 10.1371/journal.pone.0052941 23300829 PMC3534121

[251] Brooks DJ. Future imaging in dementia. Semin Nucl Med. (2021) 51:303–8. 10.1053/j.semnuclmed.2020.12.001 33353722

[252] Varley J Brooks DJ Edison P. Imaging neuroinflammation in Alzheimer's disease and other dementias: recent advances and future directions. Alzheimers Dement. (2015) 11:1110–20. 10.1016/j.jalz.2014.08.105 25449529

[253] Geevasinga N Menon P Özdinler PH Kiernan MC Vucic S. Pathophysiological and diagnostic implications of cortical dysfunction in ALS. Nat Rev Neurol. (2016) 12:651–61. 10.1038/nrneurol.2016.140 27658852

[254] Vucic S Kiernan MC. Novel threshold tracking techniques suggest that cortical hyperexcitability is an early feature of motor neuron disease. Brain. (2006) 129 (Pt. 9):2436–46. 10.1093/brain/awl172 16835248

[255] Menon P Geevasinga N Yiannikas C Howells J Kiernan MC Vucic S. Sensitivity and specificity of threshold tracking transcranial magnetic stimulation for diagnosis of amyotrophic lateral sclerosis: a prospective study. Lancet Neurol. (2015) 14:478–84. 10.1016/S1474-4422(15)00014-9 25843898

[256] Higashihara M Pavey N van den Bos M Menon P Kiernan MC Vucic S. Association of cortical hyperexcitability and cognitive impairment in patients with amyotrophic lateral sclerosis. Neurology. (2021) 96:e2090–7. 10.1212/WNL.0000000000011798 33827958

[257] Shimizu T Bokuda K Kimura H Kamiyama T Nakayama Y Kawata A. Sensory cortex hyperexcitability predicts short survival in amyotrophic lateral sclerosis. Neurology. (2018) 90:e1578–87. 10.1212/WNL.0000000000005424 29602913

[258] Blain-Moraes S Mashour GA Lee H Huggins JE Lee U. Altered cortical communication in amyotrophic lateral sclerosis. Neurosci Lett. (2013) 543:172–6. 10.1016/j.neulet.2013.03.028 23567743 PMC3716378

[259] Dukic S McMackin R Buxo T Fasano A Chipika R Pinto-Grau M. Patterned functional network disruption in amyotrophic lateral sclerosis. Hum Brain Mapp. (2019) 40:4827–42. 10.1002/hbm.24740 31348605 PMC6852475

[260] Ricci C Marzocchi C Battistini S. MicroRNAs as biomarkers in amyotrophic lateral sclerosis. Cells. (2018) 7:E219. 10.3390/cells7110219 30463376 PMC6262636

[261] Wang L Zhang L. Circulating MicroRNAs as diagnostic biomarkers for motor neuron disease. Front Neurosci. (2020) 14:354. 10.3389/fnins.2020.00354 32372911 PMC7177050

[262] Boxer AL Gold M Feldman H Boeve BF Dickinson SL-J Fillit H. New directions in clinical trials for frontotemporal lobar degeneration: methods and outcome measures. Alzheimers Dement. (2020) 16:131–43. 10.1016/j.jalz.2019.06.4956 31668596 PMC6949386

[263] Josephs KA Mackenzie I Frosch MP Bigio EH Neumann M Arai T. LATE to the PART-y. Brain. (2019) 142:e47. 10.1093/brain/awz224 31359030 PMC6736234

[264] Tomé SO Vandenberghe R Ospitalieri S Van Schoor E Tousseyn T Otto M. Distinct molecular patterns of TDP-43 pathology in Alzheimer's disease: relationship with clinical phenotypes. Acta Neuropathol Commun. (2020) 8:61. 10.1186/s40478-020-00934-5 32349792 PMC7189555

[265] Koga S Parks A Kasanuki K Sanchez-Contreras M Baker MC Josephs KA. Cognitive impairment in progressive supranuclear palsy is associated with tau burden. Mov Disord. (2017) 32:1772–9. 10.1002/mds.27198 29082658 PMC5732021

[266] Koga S Kouri N Walton RL Ebbert MTW Josephs KA Litvan I. Corticobasal degeneration with TDP-43 pathology presenting with progressive supranuclear palsy syndrome: a distinct clinicopathologic subtype. Acta Neuropathol. (2018) 136:389–404. 10.1007/s00401-018-1878-z 29926172 PMC6309287

[267] McAleese KE Walker L Erskine D Thomas AJ McKeith IG Attems J. TDP-43 pathology in Alzheimer's disease, dementia with lewy bodies and ageing. Brain Pathol. (2017) 27:472–9. 10.1111/bpa.12424 27495267 PMC8029292

[268] Nakashima-Yasuda H Uryu K Robinson J Xie SX Hurtig H Duda JE. Co-morbidity of TDP-43 proteinopathy in lewy body related diseases. Acta Neuropathol. (2007) 114:221–9. 10.1007/s00401-007-0261-2 17653732

[269] Nelson PT Dickson DW Trojanowski JQ Jack CR Boyle PA Arfanakis K. Limbic-predominant age-related TDP-43 encephalopathy (LATE): consensus working group report. Brain. (2019) 142:1503–27. 10.1093/brain/awz186 31039256 PMC6536849

[270] Buciuc M Whitwell JL Baker MC Rademakers R Dickson DW Josephs KA. Old age genetically confirmed frontotemporal lobar degeneration with TDP-43 has limbic predominant TDP-43 deposition. Neuropathol Appl Neurobiol. (2021) 47:1050–9. 10.1111/nan.12727 33969528 PMC8576062

[271] Giannini LAA Peterson C Ohm D Xie SX McMillan CT Raskovsky K. Frontotemporal lobar degeneration proteinopathies have disparate microscopic patterns of white and grey matter pathology. Acta Neuropathol Commun. (2021) 9:30. 10.1186/s40478-021-01129-2 33622418 PMC7901087

[272] Caso F Mandelli ML Henry M Gesierich B Bettcher BM Ogar J. In vivo signatures of nonfluent/agrammatic primary progressive aphasia caused by FTLD pathology. Neurology. (2014) 82:239–47. 10.1212/WNL.0000000000000031 24353332 PMC3902758

[273] Chang JL Lomen-Hoerth C Murphy J Henry RG Kramer JH Miller BL. A voxel-based morphometry study of patterns of brain atrophy in ALS and ALS/FTLD. Neurology. (2005) 65:75–80. 10.1212/01.wnl.0000167602.38643.29 16009889

[274] Whitwell JL Avula R Senjem ML Kantarci K Weigand SD Samikoglu A. Gray and white matter water diffusion in the syndromic variants of frontotemporal dementia. Neurology. (2010) 74:1279–87. 10.1212/WNL.0b013e3181d9edde 20404309 PMC2860485

[275] Murley AG Coyle-Gilchrist I Rouse MA Jones PS Li W Wiggins J. Redefining the multidimensional clinical phenotypes of frontotemporal lobar degeneration syndromes. Brain. (2020) 143:1555–71. 10.1093/brain/awaa097 32438414 PMC7241953

[276] Mecca AP Chen M-K O'Dell RS Naganawa M Toyonaga T Godek TA. In vivo measurement of widespread synaptic loss in Alzheimer's disease with SV2A PET. Alzheimers Dement. (2020) 16:974–82. 10.1002/alz.12097 32400950 PMC7383876

[277] Chen M-K Mecca AP Naganawa M Finnema SJ Toyonaga T Lin S. Assessing synaptic density in Alzheimer disease with synaptic vesicle glycoprotein 2a positron emission tomographic imaging. JAMA Neurol. (2018) 75:1215. 10.1001/jamaneurol.2018.1836 30014145 PMC6233853

[278] Jack CR Bennett DA Blennow K Carrillo MC Feldman HH Frisoni GB. A/T/N: an unbiased descriptive classification scheme for Alzheimer disease biomarkers. Neurology. (2016) 87:539–47. 10.1212/WNL.0000000000002923 27371494 PMC4970664

